# Supplementary material for: Stakeholders’ views and perspectives on treatments of visceral leishmaniasis and their outcomes in HIV-coinfected patients in East Africa and South-East Asia: A mixed methods study
Source: PLoS Negl Trop Dis. 2022 Aug 15;16(8):e0010624. doi: 10.1371/journal.pntd.0010624 (PMC9410553; doi:10.1371/journal.pntd.0010624)
Supplement: S1 Appendix — (DOCX) [file pntd.0010624.s001.docx]

**S1 Appendix:** Survey questionnaire

1. **Your background**
2. Please let us know to which of the following groups you belong (choose one):
   1. Care provider to a person co-infected with HIV and visceral leishmaniasis
   2. Physician
   3. Nurse
   4. Representative of a national program
   5. Policy maker
   6. Academic researcher
   7. Regional Technical Advisory Group member
   8. Other: _________________________________
3. Your main organization is (choose only one):
   1. Governmental organization
   2. Non-governmental organization (NGO)
   3. Academic institution
   4. Private for-profit organization
   5. International intergovernmental organization
   6. Other organization: ______________________
4. Your country: _______________________
5. Your highest attained educational degree
   1. Certificate or diploma
   2. Bachelor's degree (e.g. BSc)
   3. Master's degree (e.g. MSc, MA)
   4. Doctoral degree (MD, PhD or equivalent)
   5. None of the above
6. Your gender:
   1. Female
   2. Male
   3. Other
7. Your age:
   1. 18 – 30
   2. 31 – 50
   3. 51 – 64
   4. > 64
8. **Valuation of the outcomes of interest**
9. While all outcomes of treatment are important, people’s perceptions of their importance may vary. From your perspective, how important are the following outcomes of the treatment of visceral leishmaniasis in persons infected with both HIV and visceral leishmaniasis?

| **Outcome** | **Not important** | | | **Important** | | | **Critical** | | |
| --- | --- | --- | --- | --- | --- | --- | --- | --- | --- |
| 1. Decreasing death | **1** | 2 | 3 | 4 | **5** | 6 | 7 | 8 | **9** |
| 1. Increasing clinical cure at the time of completion of treatment | **1** | 2 | 3 | 4 | **5** | 6 | 7 | 8 | **9** |
| 1. Increasing clinical cure 6 months after completion of treatment | **1** | 2 | 3 | 4 | **5** | 6 | 7 | 8 | **9** |
| 1. Decreasing relapse (recurrence of the disease) | **1** | 2 | 3 | 4 | **5** | 6 | 7 | 8 | **9** |
| 1. Decreasing adverse events | **1** | 2 | 3 | 4 | **5** | 6 | 7 | 8 | **9** |
| 1. Decreasing serious adverse events^[[1]](#footnote-1)^ | **1** | 2 | 3 | 4 | **5** | 6 | 7 | 8 | **9** |
| 1. Decreasing complications | **1** | 2 | 3 | 4 | **5** | 6 | 7 | 8 | **9** |
| 1. Increasing patient satisfaction | **1** | 2 | 3 | 4 | **5** | 6 | 7 | 8 | **9** |

1. **Treatment options under consideration**

**East Africa**

- Combination therapy: Co-administration of infusion of liposomal amphotericin B (30 mg/kg body weight total dose: intravenous infusion of 5 mg/kg on day 1,3,5,7,9,11) and oral miltefosine (100mg in two divided doses (i.e. 50mg capsules twice) every day during 28 days).
- Monotherapy: infusion of liposomal amphotericin B only (40 mg/kg body weight total dose: Intravenous infusion of 5 mg/kg on day 1 to 5, 10, 17, 24).
- Efficacy at day 29 (cure defined as parasitological clearance at day 29): The results in a recent study show that the adjusted efficacy was 50% for liposomal amphotericin B monotherapy and 67% for the combination therapy of liposomal amphotericin B and oral miltefosine. No major safety concerns about the combination therapy were identified.
- Efficacy at day 58 (cure defined as parasitological clearance at day 58): Those who were not cured (detectable parasites) at day 29 and receiving another round of treatment (extended treatment), the adjusted efficacy was 55% for the liposomal amphotericin B monotherapy and 88% for the combination therapy.

**South-East Asia**

- Combination therapy: Co-administration of infusion of liposomal amphotericin B (30 mg/kg body weight total dose: intravenous infusion of 5 mg/kg on day 1,3,5,7,9,11) and oral miltefosine (100mg in two divided doses (i.e. 50mg capsules twice) every day during 14 days).
- Monotherapy: infusion of liposomal amphotericin B only (40 mg/kg body weight total dose: Intravenous infusion of 5 mg/kg on day 1-4, 8, 10, 17, 24).
- Efficacy at day 210 (cure defined as patient being alive and disease free (defined as absence of signs and symptoms of VL or if symptomatic, a negative parasitology by tissue aspirate at day 210): The results in a recent study (unpublished) show that the efficacy is 85% in liposomal amphotericin B monotherapy and 96% in combination therapy.
- Relapse-free survival at 12 months (patient alive and disease-free (defined as absence of signs and symptoms of VL or if symptomatic, a negative parasitology by tissue aspirate
- at day 390): 81% in liposomal amphotericin B monotherapy and 85% in combination therapy.

Notes:

- Liposomal amphotericin B is given as slow intravenous infusion (up to 4 hours) and requires hospitalization. It requires storage in a cold chain (2-25 degree centigrade). It is contraindicated in those patients who have demonstrated or have a known hypersensitivity to amphotericin B deoxycholate or any other constituents of the product.
- Oral miltefosine often causes diarrhea and is contraindicated in pregnant women. Therefore, oral miltefosine prescribing requires mandatory pregnancy screening and use contraception in all females of child bearing age, during the treatment and at least 3 months after the treatment. It is also contraindicated in patients showing hypersensitivity to miltefosine or any of its excipients and in patients with Sjogren-Larssson-Syndrome
- The duration of hospitalization depends upon the associated morbid conditions e.g. TB and other opportunistic infections but it is significantly less in combination therapy than monotherapy in both East Africa and South-East Asia.
- Both the drugs are currently in use in VL programmes and the standard administration and follow-up of patients will remain the same.

1. **View about the treatment options under consideration**

**Can you please rate combination therapy as compared to monotherapy with relation to the following?**

**Equity**

1. What would be the impact of combination therapy (as compared to monotherapy) on health equity (every coinfected patient has a fair and just opportunity for receiving the appropriate treatment)?
   1. Reduced health equity
   2. Probably reduced health equity
   3. Probably no impact on health equity
   4. Probably increased health equity
   5. Increased health equity
   6. Varies; explain (under comments section):
   7. Don't know

Comments: ______________________

**Acceptability**

1. Health care provider’s acceptability: Is combination therapy (as compared to monotherapy) more acceptable for the health care provider?
   1. Not acceptable
   2. Probably not acceptable
   3. Probably yes, acceptable
   4. Yes acceptable
   5. Varies; explain (under comments section):
   6. Don't know

Comments: ______________________

**Feasibility**

Note: this may include aspects of offering combination therapy and treatment of HIV-VL services at district hospitals or non-specialized hospitals with a need for more skilled staff, more logistic requirements and others.

1. Is combination therapy (as compared to monotherapy) more feasible to implement in district hospitals or non-specialized centers?
   1. Not feasible
   2. Probably not more feasible
   3. Probably yes, more feasible
   4. Yes, more feasible
   5. Varies; explain (under comments section):
   6. Don't know

Comments: ______________________

**Implementation**

1. What are specific implementation considerations relevant to either combination therapy or monotherapy?

Comments: ______________________

**Monitoring and evaluation**

1. What are specific treatment-monitoring and evaluation considerations relevant to either combination therapy or monotherapy? Note: Are indications or contraindications be managed and monitored as easily in combination therapy as in monotherapy? Other considerations?)

Comments: ______________________

**Research priorities**

1. What are specific research priorities for the treatment of VL in HIV-VL coinfected persons?

Comments: ______________________

**Additional comments**

1. Is there anything else that you think is important for the treatment of VL in HIV-VL coinfected persons that has not been mentioned?

Comments: ______________________

1. A **serious adverse event** (**SAE**) is defined as any untoward medical occurrence that at any dose results in death or is life-threatening or requires inpatient hospitalization or causes prolongation of existing hospitalization or results in persistent or significant disability/incapacity or may have caused a congenital anomaly/birth defect, or requires intervention to prevent permanent impairment or damage [↑](#footnote-ref-1)
