# Supplementary material for: Stakeholders’ views and perspectives on treatments of visceral leishmaniasis and their outcomes in HIV-coinfected patients in East Africa and South-East Asia: A mixed methods study
Source: PLoS Negl Trop Dis. 2022 Aug 15;16(8):e0010624. doi: 10.1371/journal.pntd.0010624 (PMC9410553; doi:10.1371/journal.pntd.0010624)
Supplement: S3 Appendix — (DOCX) [file pntd.0010624.s003.docx]

**S3 Appendix:** Survey participants’ characteristics by region (N=177)

|  | South-East Asia (N=142)* | East Africa (N=32)* | Other/not specified (N=3)* |
| --- | --- | --- | --- |
| **Gender** |  |  |  |
| Female | 18 (13) | 8 (26) | 0 (0) |
| Male | 123 (87) | 23 (74) | 2 (100) |
| **Age (years)** |  |  |  |
| 18–30 | 22 (15) | 1 (3) | 0 (0) |
| 31−50 | 90 (63) | 23 (72) | 2 (67) |
| 51−64 | 22 (15) | 8 (25) | 1 (33) |
| > 64 | 8 (6) | 0 (0) | 0 (0) |
| **Highest attained educational degree** |  |  |  |
| Doctoral degree | 49 (35) | 14 (45) | 2 (67) |
| Master’s degree | 51 (36) | 13 (42) | 1 (33) |
| Bachelor’s degree | 32 (23) | 3 (10) | 0 (0) |
| Certificate or diploma | 3 (2) | 1 (3) | 0 (0) |
| None of the above | 7 (5) | 0 (0) | 0 (0) |
| **Stakeholder group** |  |  |  |
| Health providers/clinical officers | 51 (36) | 11 (34) | 0 (0) |
| National NTD officers/subnational programme managers | 43 (30) | 8 (25) | 1 (33) |
| Researchers | 15 (11) | 10 (31) | 0 (0) |
| Nongovernmental organizations | 12 (8) | 1 (3) | 1 (33) |
| Regional and country office WHO staff | 4 (3) | 0 (0) | 1 (33) |
| Policy-makers | 3 (2) | 0 (0) | 0 (0) |
| Members of WHO’s Regional Technical Advisory Group on VL | 2 (1) | 0 (0) | 0 (0) |
| National ministry of health staff | 0 (0) | 1 (3) | 0 (0) |
| Not specified | 12 (8) | 1 (3) | 0 (0) |
| **Affiliation** |  |  |  |
| Nongovernmental organization | 81 (57) | 6 (19) | 1 (33) |
| Governmental organization | 29 (20) | 18 (56) | 1 (33) |
| International intergovernmental organization | 16 (11) | 4 (13) | 1 (33) |
| Academic institution | 12 (8) | 4 (13) | 0 (0) |
| Private for-profit organization | 2 (1) | 0 (0) | 0 (0) |
| Not specified/other | 2 (1) | 0 (0) | 0 (0) |

*Numbers do not always add to totals due to missing data. Percentages represent valid percentages.

Abbreviations: NTD: neglected tropical diseases; WHO: World Health Organization; VL: visceral leishmaniasis
