# Supplementary material for: Stakeholders’ views and perspectives on treatments of visceral leishmaniasis and their outcomes in HIV-coinfected patients in East Africa and South-East Asia: A mixed methods study
Source: PLoS Negl Trop Dis. 2022 Aug 15;16(8):e0010624. doi: 10.1371/journal.pntd.0010624 (PMC9410553; doi:10.1371/journal.pntd.0010624)
Supplement: S4 Appendix — (DOCX) [file pntd.0010624.s004.docx]

**S4 Appendix:** Detailed presentation of the findings from the semi-structured interviews organized by construct (Tables A-E)

***Note:*** *‘P’ refers to patients and ‘NP’ refers to non-patient stakeholders*

**Table A: Valuation of the outcomes of interest**

| **Outcome** | **Valuation of the outcome** | **Illustrative quotes** |
| --- | --- | --- |
| Mortality | Reducing mortality is critical for any disease (NP) | *“It is definitely important because the life of every person is important.”* (India, NP9)  *“I mean for any program, mortality has always been critical indicator.”* (Bangladesh, NP3) |
|  | Survival considered as a miracle (P) | *“I lost faith that I will be alive, but it is like miracle that I am fine now after treatment.”* (India, P7) |
|  | Experiences of death among close relationships (P) | *“My friends are not alive. Most dead. Only few persons who correctly follow treatment are alive.”* (Ethiopia, P5) |
|  | Increased vulnerability and faster disease progression due to coinfection and immunosuppression (NP) | *“VL can fasten the progress of HIV disease, so it can fasten the mortality of those HIV patients, so VL treatment are very important and decreasing mortality rate for those patients, so it is a very important outcome.”* (Ethiopia, NP6)  *“No patient dies from VL, patients die from suppressed immunity, coinfections, anemia, and so on.”* (India, NP7) |
| Disease-related complications | Irreversible (NP) | *“Complications also is a very important outcome that we have to focus on starting from the beginning because if those patients develop complications once, sometimes it might not be reversible, so we have to give focus or an emphasis from the beginning on what the complication is.”* (Ethiopia, NP6) |
|  | Life-threatening (NP) | *“HIV-VL is a life-threatening disease, it is not like any other diseases. So when the parasite enters into the body, the spleen is getting enlarged […] and complications like dehydration, malnutrition […] will be very life-threatening”* (India, NP10) |
|  | Has financial implications (NP) | *“I mean, if patient suffers for long time, from many complications that has many implications, for example financial implications, or health system will be suffered as well.”* (Bangladesh, NP3) |
|  | An expected outcome of the disease (NP) | *“They should be expected. Complications should be expected as a norm rather than the exception.”* (India, NP7) |
|  | An objective of being treated (NP) | *“It is also important. For any patient, the objective is that fully cure, if not possible, at least decreasing complications.”* (India, NP9) |
|  | Less important than relapse and cure (NP) | *“Decreasing complications, also important but […] the first few parts are more important […] but maybe we can go with some complications, even, if there are no relapses and it’s treated.”* (India, NP2) |
| Clinical cure | Opportunity to go back to daily life activities and be productive (P and NP) | *“When I completed treatment and discharged, I am healthy working and eating well. [...] I am working, walk long distance when I am free from Kalazar.”* (Ethiopia, P5)  *“I started doing my household work after treatment which I was not able to do when I was sick.”* (India, P2)  *“It is very important from patient perspective, because ultimately that person has to lead a socially and economically productive life.”* (India, NP9) |
|  | Importance of long-term over short-term cure (NP) | *“The main problem of treating HIV-VL is that the initial cure is 100% with any therapy, you give miltefosine only, you give paromomycin only, or liposomal amphotericin B, conventional amphotericin B, the initial will be the same, 100% cure rate. But in the long term, if you follow the patient, the patient will relapse and relapse and relapse”* (India, NP8)  *“Just after completion of the treatment it will be the preliminary outcome and the final outcome will be after 6 months.”* (Bangladesh, NP3) |
| Relapse | Common (NP) | *“So, whatever arm of treatment we use, the relapse rate has to be decreased, because the relapse rate in HIV-VL patients is much more than a common VL patient.”* (India, NP5)  *“In co-infected patients, the general rule is that they will relapse unless their immunity picks up, knowing that it takes quite a long time for immunity to pick up.”* (India, NP7) |
|  | Risk of recurrent relapses (NP) | *“But in the long term, if you follow the patient, the patient will relapse and relapse and relapse”* (India, NP8)  *“So, relapse is also one of the important outcomes for those kinds of patients like HIV, especially for those treated 2-3 times, we anticipate that they will come in the coming few months, even here we had 17 up to 35 times relapse cases for one patient.”* (Ethiopia, NP6) |
|  | Raises doubts about treatment effectiveness and affects patients’ mental health (NP) | *“It is psychologically very depressing for them. It raises questions in their mind: why are we taking treatment? It raises a question in their minds that this treatment is not effective... Overall, thinking like that, hopeless case, nothing will happen to this patient, and they have already psychosocial burden of the HIV. It is doubled and it is a big issue today.”* (India, NP9) |
|  | Increases patient suffering (NP) | *“If the patient develops relapse, then again he has to go through with all these procedures and in sufferings.”* (Bangladesh, NP3) |
|  | Increases risk of mortality risk (NP) | *“And relapse, relapse, relapse, is the main problem. Relapse, morbidity, and ultimately death.”* (India, NP8) |
|  | Increases risk of resistance and treatment failure (NP) | *“For the treatment part, physicians, because as the patient suffers more and more episodes, chances are that the drug might not work, or there might be some sort of resistance after multiple exposures”* (India, NP2)*.* |
|  | Increases risk of disease transmission (NP) | *“[the patients is an] important reservoir for the infection, so he can spread to the other person. So the minimum the relapse, it’s better for everyone.”* (India, NP2)  *“Because the relapse is bad for the patients and for the society as well, because they are spreading, they are the highest killers”* (India, NP5) |
| Non-serious side effects | Well tolerated and easily treated (NP) | *“Nausea, not much problem, we can treat it easily.”* (India, NP8)  *“With AmBisome it is at the time of infusion only, most of the time... So these are very tolerated by the patient. For the miltefosine, as long as the patient takes the treatment, there are chances of vomiting, all these things, but they can also be managed with drugs. So among these two serious and, so, I would say the serious would be minimum.”* (India, NP2) |
|  | An expected outcome of the treatment (NP) | *“Common side effects are to be expected. It is interesting to monitor them just from a scientific perspective”* (India, NP7) |
| Serious side effects | Additional burden on patients (NP) | *“The serious side effects, they are very serious, as the name indicates, so the side effects are very important thing that we have to notice and consider in those patients, because those patients are like a glass, because their immunity is too decreased by HIV, so we have to be careful at the side effects, so looking on side effects is one of the very important indicators or parameters on HIV patients. We are worried about side effects. So yes, I give you high emphasis on it as a parameter.”* (Ethiopia, NP6) |
|  | Additive effect with other treatments’ side effects (NP) | *“Because there may be additive toxicity with ART and combination therapy. Amphotericin B is nephrotoxic, tenofovir in ART is also nephrotoxic. So, […] toxicity is one of the important factors for therapy.”* (India, NP8) |
|  | Increases risk of permanent damage (NP) | *“And then of course if you want me to score all this, I’ll put serious adverse events after mortality, because mortality and some permanent damage, these are, I mean these can actually happen after treatment, so after mortality, in my opinion, serious adverse events should come after mortality when I talk about importance of all this.”* (Bangladesh, NP3) |
|  | Increases risk of mortality (NP) | *“In serious side effects, you have to be very careful because then the mortality rate can be high with serious side effects.”* (India, NP3) |
| Patient satisfaction | Critical for any disease, but overlooked (NP) | *“I think it is critical. We do not look at it normally.”* (India, NP7)  *“The patients need to be satisfied at the end of treatment, especially.”* (India, NP6)  *“So, patient satisfaction is one of the very important outcomes at the end. Actually, here we are not assessing at the end of treatment, we observe them by asking some questions are you satisfied or not. It is an important indicator especially as a protocol-based or as documentation-based, as parameter.”* (India, NP6)  *“No, not in this population, for every population, for every person, even you… me… When we have some disease, we are getting treatment, we want to be cured, we want to be satisfied with our health. They are not a special entity or something.”* (India, NP9) |
|  | Contributes to patient’s faith in the health system (NP) | *“This [patient satisfaction] is very important. I would say. Because the faith in health system by the patient is far most important”* (India, NP2)  *“So if they are satisfied, then like even they will go with the other patient, they can say that like this treatment works and improves the quality of life. So most of the time it’s less people die.”* (India, NP2) |
|  | Driven by care and improvement (P) | *“With the treatment and immediate improvement we are satisfied.”* (Ethiopia, P1)  *“Especially, [patients] are satisfied based on the clinician’s approach. If you approach them very nicely, just asking practically do you feel better, they will be more satisfied.”* (India, NP6) |
|  | Less important than cure (NP) | *“Patient satisfaction I think it will maybe come at the end, because we don’t have many options for treatment of HIV and VL patients, so the scopes, I mean the options are limited, and both are really serious diseases, so I think patients would be more concerned about their progress and improvement rather than mild dissatisfaction.”* (Bangladesh, NP3) |

**Table B: Impact on equity**

| **Equity issue** | **Views** | **Illustrative quotes** |
| --- | --- | --- |
| ***Equity issues related to combination therapy*** | | |
| No access issue with combination therapy | Both alternatives currently available free of cost through government support and donations (P and NP) | *“For economic, I would say that treatment is absolutely free in India.”* (India, NP1)  *“All the drugs are provided by the government, so the financial or other issues are not, these are not actually of concern here.”* (Bangladesh, NP3)  *“Treatment is given free of cost, so there is no question of economic status.”* (India, NP8)  *SKI05: “Cost is covered by the government. Patients are getting both treatments for free.”* (India, NP5)  *“Yes, no problem. Everybody can get the service without any economic issue.”* (Ethiopia, NP6)  *“The doctors are treating properly, and the treatment is free of charge I am happy. It is very beneficial for economic aspect.”* (Ethiopia, P1) |
|  |  | *“But as always there is cost issue for those, they are just supplying by donations, and plus this miltefosine is a new product for our country, and even for the VL program, we are just access for the products.”* (Ethiopia, NP4)  *“It is the responsibility of the government, in most countries, to ensure that all of its citizens will get medicine, best medicine, as far as possible […] The guideline should advocate it (more effective treatment), this is the best treatment, this is the costing issue, and the government will take the decision, less effective treatment based on the cost, again the government should have an informed decision … It should be clear.”* (India, NP9) |
|  | Both alternatives require hospitalization (NP) | *“For both cases the patients will have to be hospitalized, so I think this will not be an issue.”* (Bangladesh, NP3) |
|  | Equal access for different geographical, gender and age groups (NP) | *“I think this combined therapy just meets equity for the patient, […] this combined therapy can just have good equity perspective for those patients who are HIV-VL co-infected.... It is possible just implement the combination therapy regardless of the geographical or the other demographic difference or gender difference and age.”* (Ethiopia, NP4) |
| Pregnant women and women of childbearing age who may not prevent pregnancy not eligible | - | *“Combination therapy may have equity issues because pregnant women cannot receive this medication... I mean consider combination therapy as first line and then for those who cannot actually receive combination therapy then for them they may receive the second line, maybe monotherapy.”* (Bangladesh, NP3)  *“Combination therapy is typical to give in females of reproductive age group. So if we are treating there is problem with this drug cannot be given in females of reproductive age who may not prevent pregnancy […] So combination therapy is more acceptable but combination therapy is not equitable, you cannot give it to all the patients. You cannot give it irrespective of age, this is the problem”* (India, NP8) |
| ***Equity issues not related to combination therapy but relevant to patients with HIV-VL in general*** | | |
| Access to treatment centers | Long distance to centers (NP) | *“It might be somewhere like far from the patient, around 10 or 15 miles”* (India, NP2)  *“Accessibility is a very big issue in India, that they even have to travel 200 kilometers, something like that. Accessibility, sometimes in a state there may be only one center. For treatment people have to come around 200-250 kilometers.”* (India, NP9) |
|  | Limited number of centers (NP) | *“There is need to increase accessibility by increasing the number of treatment centers and there are tertiary care centers that can provide this thing.”* (India, NP9)  *“There are four states in India which have HIV/VL services. The state with the highest number of cases has only one center for HIV/VL treatment, so this will create challenges of travel, etc. But it is a very good institution, many times they are paid for the travel (this was the case, not sure this is still happening.”* (India, NP5)  *“Co-infected patients have to move to a higher health center, if he’s staying at a village level, so he has to move onto a higher health center. […] Because each state has got many districts, so at the district level [patients with VL] can get access to VL treatment with liposomal amphotericin B at the primary level, but for HIV VL has to come up to a health center either secondary, or sometime patients might consider it to be difficult to handle and they send it to a tertiary care level.”* (India, NP1) |
|  | Limited centers providing specialized care (NP) | *“I do think it is critical that co-infected patients are treated in appropriate centers of care, treatments are long, complicated, there is a high number of outcomes for serious side effects, there are multiple disciplines that need to be involved and these just simply are not available. There are a few of them. […] We know that these patients, generally speaking, have got CD4 count of less than 200, 80% of them have CD4 count of less than 200, which means they are indicated to have CRAG test as well, extra screening for TB, all these things, ultrasound, screening for abdominal TB, these are important to have […] but these things are not available in most facilities, even in the center.”* (India, NP7) |
| Access to treatment | Limited supply of treatment (NP) | *“Especially in the government side, they are creating some equity issues, because of a supply problem. There is no well supply in the government, so government hospitals are locked down of giving service and referring them to MSF hospitals. […] This started since November- December 2019 (sending patients to MSF). When we ask them, they do not have AmBisome and miltefosine especially AmBisome because they cannot access them easily. So, they are sending the more complicated cases to MSF. Actually, it might not go with the social, but it seems somehow an economical issue.”* (Ethiopia, NP6)  *“Currently both drugs are available, but I guess liposomal amphotericin B is probably in a very small number of stock.”* (India, NP1)  *“The limitation would be just accessibility for the drugs (miltefosine)... I mentioned some issues concerning availability.”* (Ethiopia, NP4) |
|  | Lack of physician awareness of available treatments in non-VL endemic areas (NP) | *“Doctors practicing elsewhere in the state, where VL is not endemic, they really don’t know about such things.”* (India, NP1) |
| Economic impact | - | *“But from the difference in treatment, I think the same thing goes for both groups, both need to be ideally treated away from home for the shortest period of time, if it is safe for them to be so, in order to reduce wage losses, keeping in mind that the multi-disciplinary approach of treating these coinfected patients is something that I think is quite expensive to be done, it can’t be afforded by the typical patients, outside the specialized centers.”* (India, NP7) |
| Gender | Worse access to the health system for women (NP) | *“Males have always better access to health system as compared to women.”* (India, NP1) |
| HIV testing | Low HIV testing justified by low HIV caseload (NP) | *“They do not have a very big HIV caseload, so they are not doing it. But the thing is that it may be possible, if the HIV caseload is very low, one or two patients may miss the proper treatment. That is also not equitable. Country has kit for testing HIV, country has everything for treatment of HIV/VL coinfected patients but based on their belief that they do not have HIV caseload, they do not do HIV testing, that is not right […] When we are not doing the HIV testing, we may miss the opportunity to give a proper treatment. It should be mandatory in every country.”* (India, NP9) |
| Access to information | Limited access to information (NP) | *“In my opinion, if we want to ensure equity, we must provide them with proper information. If we want to ensure equity in the treatment of HIV/VL, we first need strong evidence, it should not be that we impose that it is less important so you should not use it... Every community member should have that information. Equity in information. Equity in treatment, you have to answer equity on information, the right to have that information... If … treatment is better, so they have the right to choose, they can select.”* (India, NP9) |
| Stigma | HIV status (NP) | *“Stigma lies with HIV. They are afraid of showing their antiretroviral drugs for their neighbors.”* (India, NP1)  *“So in our community, long duration of inpatient treatment or management is just not acceptable, just because there may be exposure for those of their confidentiality, because HIV is sensitive for the stigmatization.”* (Ethiopia, NP4)  *“Actually, what I want to stress from the very beginning, they are from a poor community, they are stigmatized... If there is less effective treatment, but there is this positive thing, that listening to stigmatized patients.”* (India, NP9) |
| Attitudes related to seeking healthcare | Initial seeking of healthcare in private hospitals and spending considerable amount of money (P) | *“I spent huge money (appx. - 60000 INR) in private hospitals before getting for the actual treatment.”* (India, P1)  *“No wage loss provided for my treatment like other kala-azar cases. I spent huge money with private hospitals. It was around 10 000 INR (apx. 130 USD)”* (India, P5)  *“I spent huge amount of money (INR 50000; USD 670) for my treatment in private hospitals and had travelled to various places for treatment.”* (India, P7)  *“I spent huge money (appx. – 220000 INR, USD 3000) in private hospitals before getting for the actual treatment.”* (India, P8)  *“I spent huge money (appx. – 35 000 INR, USD 400) in private hospitals before getting treatment in government hospital. No money was asked for kala-azar treatment.”* (India, P10) |

**Table C: Feasibility of combination therapy in relation to monotherapy**

| **Determinants** | **Consequences** | **Favors** | **Illustrative quotes** |
| --- | --- | --- | --- |
| Shorter duration of hospitalization with combination therapy | Decreased economic burden on the health system (NP) | Combination therapy | *“Long hospitalization may request more cost than short period of hospitalization so it is so feasible than the monotherapy.”* (Ethiopia, NP4) |
|  | Decreased economic burden on the patient (P and NP) | Combination therapy | *“36 days is a very long period of time to manage these patients as in-patients considering the economic cost and economic burden of loss of wages for these patients.”* (India, 7)  *“So economically, this is a big burden for the patient because the patient has to stay.”* (India, NP5)  *“Staying for shorter is convenient for me as I can save time, save money”* (India, P9) |
|  | Decreased burden on accompaniment (P and NP) | Combination therapy | *“In that case, the question of economic status comes, because usually patients are accompanied by one or two persons from the family, and they have to maintain them in the town for treatment, and it is not feasible for them to stay so long along with the patient in the town for treatment. So economic situation also comes here. It will be difficult for them to do accompaniment in town or city where they receive the treatment […] Most of the patients are from very remote rural area, so monotherapy is less feasible in this case. As they cannot stay long in the hospital, far from his village, with some accompaniment.”* (India, NP8)  *“Shorter duration of hospitalization is more feasible to me as it will be convenient for me and as well my family and attendants.”* (India, P10) |
|  | Being more able to cater to daily responsibilities (P) | Combination therapy | *“Shorter duration of hospitalization is more feasible to me as I can come back my home and take care of house work and children”* (India, P2) |
| Additional administration of oral drug as part of combination therapy | Treatment is finished at the hospital (NP) | Either | *“They are not taking medicine on their own, the nurse is giving it to them. […] Yea yea yea, you have to understand, that for one to 11 days, the patient is in hospital taking AmBisome for the combination and maybe for one or two days the patient is there for the rest of the treatment. So, the treatment is almost finished in the hospital, so they are not taking their medicine on their own, the nurse is giving them their medicine.”* (India, NP5) |
| Contraindication in pregnancy of combination therapy | Difficulty with counseling (NP) | Monotherapy | *“For combination therapy, the explaining part is a little bit difficult. If she is a female patient, need to consider if she is married or not and cannot get pregnant. Otherwise, both are the same.”* (India, NP5) |
|  | - | Monotherapy | *“Combination therapy is typical to give in females of reproductive age group. So if we are treating there is problem with this drug cannot be given in females of reproductive age who may not prevent pregnancy. Just married women, and married women. It is unlikely in our country that a single lady to get pregnant. But married lady, and pregnant lady, the combination therapy cannot be given, so it is very difficult to practice this therapy. It is not feasible to give this drug therapy to these persons.”* (India, NP8) |

**Table D: Acceptability of combination therapy in relation to monotherapy**

| **Determinants** | **Consequences** | **Favors** | **Illustrative quotes** |
| --- | --- | --- | --- |
| Shorter duration of hospitalization with combination therapy | Less economic burden on the patient (NP) | Combination therapy | *“So economically, this is a big burden for the patient because the patient has to stay, an attending has to stay, so loss of wages, a lot of this type of waste. So, in that manner, the combination therapy is better, because the patient has to stay for 11 days, maybe 15 or 16 days.”* (India, NP5)  *“Regarding acceptability, the combination therapy will be more acceptable, more acceptable. Because hospital stay is less. And in our country, most of the persons are from working class, very poor [...] So it is very difficult for them to get admitted in a big hospital, or to stay for one month or longer, for two months. So combination therapy is more acceptable.”* (India, NP8) |
|  | Less burden on family and caregivers (P and NP) | Combination therapy | *“Caregivers are also affected because caregivers are attendants with patients. So, I do think it important to have a lower duration of treatment.”* (India, NP7)  *“The economic loss is not only for the patient; it is for the attending as well.”* (India, NP5)  *“Shorter duration of hospitalization is more acceptable to me as it is very difficult to stay in hospital for family and attendants.”* (India, P8)  *“Shorter duration of hospitalization is more acceptable as it won’t burden on my attendants who has to visit and take care of my food.”* (India, P10) |
|  | Less risk of loss of confidentiality (NP) | Combination therapy | *“So in our community, long duration of inpatient treatment or management is just not acceptable, just because there may be exposure for those of their confidentiality, because HIV is sensitive for the stigmatization.”* (Ethiopia, NP4) |
|  | Increased comfort (P and NP) | Combination therapy | *“They may not have comfortable to just admitted in the hospital and to treat for long period. Rather, they need just to treat as quickly as possible and to get out of their hospital or facility.”* (Ethiopia, NP4)  *“Sanitation and food is good at home.”* (Ethiopia, P4)  *“Short duration is preferred… For me the long duration stay in hospital is not comfortable… Short hospital stays and follow the remaining at home.”* (Ethiopia, P9)  *“Two drug is more acceptable as it will require short duration of hospital stay. I can go home and can take rest and good food. Home is home and home environment are always good.”* (India, P9) |
|  | Improved bed occupancy (NP) | Combination therapy | *“Also acceptable to the, I mean, health service providers. Because we have shortage of hospital beds, so if I can reduce the hospital stay by 50%, that would be really helpful.”* (Bangladesh, NP3) |
|  | Improved safety of accompaniment in hospital environment (P) | Combination therapy | *“Shorter duration is always good if it is matter of staying in hospital as hospital is not safe place to stay for family, attendants and guest.”* (India, P7) |
|  | Less separation from family (P) | Combination therapy | *“He can return to his home soon… I do not prefer staying long in hospital. It separate family… Short hospital stays and further taking drugs at home. Because I can reassure my family.”* (Ethiopia, P6) |
|  | Less hospital-acquired infections (P) | Combination therapy | *“Short hospital stays. different additional diseases might come.”* (Ethiopia, P4)  *“Staying in hospital for short time and continue the remaining at home to avoid hospital acquired infections.”* (Ethiopia, P3) |
|  | Being more able to cater to daily responsibilities (P) | Combination therapy | “*Shorter duration of hospitalization is good if health condition improves. I can do my household chores and take care of children at home.”* (India, P2) |
|  | Less medical supervision at the hospital (P and NP) | Monotherapy | *“Tablet should be given at hospital and not at home as there would be high chance of missing doses if taken at home.”* (India, P1)  *“Taking medication in hospital is safer as doctors can take care of me if I have any side effect from tablet.”* (India, P7)  *“At hospital for doctor to monitor & treat side effects.”* (India, P3)  *“Long duration of hospitalization is more acceptable to me as more attention can be given to me.”* (India, P5)  *“Long duration of hospitalization is fine for me as doctors can give more time on me for treatment […] Long duration of hospitalization is more acceptable to me as my illness can be diagnosed well and I can be fully traded if I stay for long time in hospital.”* (India, P6)  *“For me the suitable is staying in long time hospital under observation of doctors because I may miss timing and also forget the medication and disease may worsen.”* (Ethiopia, P2)  *“I did not support taking medication at home. Because I may not follow the amount and time. I need doctors support. I did not have comfortable home set up.”* (Ethiopia, P5)  *“Stay in hospital for long time and seek professionals’ advice.”* (Ethiopia, P7) |
|  | Home being unfavorable environment for cure (NP) | Monotherapy | “*No, because those HIV patients even they are low in socio-economic status, they are malnourished, […], so they prefer even to stay in the hospital […] and they do not hesitate for long duration.”* (Ethiopia, NP6) |
|  | Duration not the priority (NP) | Either | *“The patients want to get cured at the end of treatment. That’s why they even do not worry about the stay or short duration.”* (Ethiopia, NP6) |
| Less IV administration more oral administration with combination therapy | Less fear of IV (NP) | Combination therapy | *“In AmBisome they are taking 8 doses, while in miltefosine they are taking 6 doses. There is a notion in many places in India that they are afraid of IV drip. Many tribal populations in India refuse sometimes to take any treatment because they are so afraid. So, in that way the combination is better, because they have less IV drip.”* (India, NP5) |
|  | Belief that oral medication will not cure them, prefer injection (P) | Monotherapy | *“Tablet has no role in treatment as I did not find any effect of tablet. All effect was due to injection.”* (India, P4)  *“Taking one drug, injection is more acceptable to me as I know only injection can treat this disease […] I do not prefer oral medicine as I know it will not cure my disease.”* (India, P6) |
| Patients’ belief that combination therapy is more effective | - | Combination therapy | *“Interviewer: Are you saying that patients know that it is more effective? They prefer it because it is more effective? NP6: Yes, more effective.”* (Ethiopia, NP6) |
| Contraindication in pregnancy of combination therapy | No issues with pregnancy test (P) | Either | *“Getting pregnancy test done is not issue for me and if it is required, it must be done.”* (India, P2) |
|  | No issues with use of contraceptives (P and NP) | Either | *“From a cultural perspective, I think yes, there is a general antipathy towards the use of contraceptives. However, in this specific patient group I don’t think this is the case. The patients are quite unwell and they are quite understating and willing to accept contraceptives in order to ensure that they are able to receive treatment.”* (India, NP7)  *“I have no issue in use of contraception if it is required for 3 months. However, it was not a problem for me as I am already operated for that”* (India, P3) |
|  | Pregnancy test might be a cultural issue (NP) | Monotherapy | *“For many developing counties, this becomes a bit of a challenge. For example, in India, suppose we ask an unmarried lady of 17 years to do a pregnancy test. So, in India, if we ask females to do pregnancy test, we will face a lot of problems, this is because of the culture. We do not face this type of problems with single dose therapy. […] So, in one way the acceptability is less in unmarried women, or women of childbearing age in India.”* (India, NP5)  *“For issue like that in India in unmarried women to test for pregnancy, it may create a problem.”* (India, NP9) |
|  | Desire for pregnancy among women of childbearing age (NP) | Monotherapy | *“Somehow monotherapy is more acceptable […] because some ladies they prefer to get pregnant after getting treatment […] or having plan, the long-term family plan […] The problem is that they do not want to take miltefosine because they want to get married/ get pregnant.”* (Ethiopia, NP6) |
| Side effects with combination therapy | Increased due to combination of drugs (P and NP) | Monotherapy | *“My only concern with combination therapy will be interaction of ART plus miltefosine. So if the patient, there, with the miltefosine, there might be some side effects.”* (India, NP2)  *“Combining treatments is fine. I do not think it has any practical or pragmatic implications apart from possibility of high number of side effects, that is by far outweighed by the benefits of the treatment.”* (India, NP7)  *“One drug is more acceptable as it is easy to take and tolerate one drug”* (India, P4)  *“One drug is convenient for me as body can set with one drug easily and it will be problem if two drug is given for one disease”* (India, P6)  *“Two drugs may cause side effects. It is tiresome.”* (Ethiopia, P4) |
|  | Added side effects of miltefosine (NP) | Monotherapy | *“We came across cases who had SE due to miltefosine like nausea vomiting etc. and they stop taking this medicine […], so relapses following incomplete doses of miltefosine was quite common.”* *(talking about an earlier regimen which is not practiced anymore)* (India, NP1)  *“Because miltefosine has some complications like diarrhea nausea vomiting, and we have report of discontinuation of drugs, umm with miltefosine.”* (Bangladesh, NP3) |
| Increased pill burden with combination therapy | - | Monotherapy | *“With the miltefosine, there might be some […] issue of pill burden.”* (India, NP2) |
| Doctor’s advice | - | Either | *“Drug which can cure me completely and which doctor feels should be prescribed, is best for me irrespective of it is one drug or two drugs.”* (India, P1)  *“Whichever doctor feels is good more me, that would be acceptable to me. If capsule is good and it does not affect my kidney, I am fine with two drugs […] Whatever doctor advises me, I would follow that. I prefer to take medicine at home if it will not cause any problem.”* (India, P2)  *“Both are fine for me whatever doctor will provide me and good for my health.”* (India, P8) |
| Perceived effectiveness | - | Either | *“Shorter duration of hospitalization is more acceptable to me but with condition that I should be fully recovered from my disease.”* (India, P1)  *“Shorter duration of hospitalization is good if health condition improves.”* (India, P2) |

**Table E: Implementation considerations, monitoring and evaluation, research priorities**

|  | **Illustrative quotes** |
| --- | --- |
| ***Implementation considerations*** | |
| Patient involvement (NP) | *“Having them involved and aware of what is going on and what potential risks are, and what the expected complications could be is absolutely critical.”* (India, NP7) |
| Compassion by health providers (NP) | *“They have HIV, which is more stigmatized in our country, so those patients who come to our facility and they treat by those combined therapy, just they feel like just the facility or the government or the health providers are compassionate for them.”* (Ethiopia, NP4)  *“Actually, what I want to stress from the very beginning, they are from a poor community, they are stigmatized... If there is less effective treatment, but there is this positive thing, that listening to stigmatized patients.”* (India, NP9) |
| Counselling (P and NP) | *“For me, patient satisfaction is very complicated to answer, because the patient is so irritated, that even if the clinical progression is good, the patient may not be satisfied because he has so many problems and is very very irritated. So, with the treatment, counseling is equally important.”* (India, NP5)  *“Two infections HIV and Kalazar. As to me first the person who has HIV and diagnosed are difficult so counseling, telling cause of Kalazar, how it will relapse and how to prevent relapse.”* (Ethiopia, P2) |
| Holistic and multi-disciplinary approach (NP) | *“Treatment, psychosocial, and economic needs as part of the package, if you want to give them justice and have an impact on mortality and improve outcomes […] Yes, I think outcomes with regard to other opportunistic infections at evolution of the management of HIV should be looked at both at the same time. This has to be seen as a multi-disciplinary approach and it will be directly associated with long term outcomes with regards to VL treatment and relapse is associated with that. It is possible to look at secondary outcomes with regard to other opportunistic infections and evolution of HIV disease”* (India, NP7)  *“Those HIV patients are living in low socio-economic status, so they are not well nourished at home and it has an effect on kala azar treatment that causes frequent relapses. […] So, malnutrition is one of the challenges here” (Ethiopia, NP5)* |
| Appropriate level of care (NP) | *“These patients need to be treated in a special facility that is aware of how to deal with these complications.”* (India, NP7) |
| ***Monitoring and evaluation*** | |
| Need for further clarity on monitoring (NP) | *“Actually, follow-up is very mandatory, but what follow-up means? Is it coming back to the hospital and being seen by doctor or health worker will go to the community? It should be specific, because in South Asia I have seen everything. Some patients come to the hospital and the doctor follows up with proper medical checkup. Second, I have seen some health worker goes and asks, how are you? etc. That how they follow up. So, it should be clear in the guideline, what follow-up means, whatever they have to do it.”* (India, NP9) |
| Need for monitoring of additive side effects and pharmacovigilance (NP) | *“For combination therapy, we have to monitor for side effects of two drugs, and there is also additive side effects, like Amphotericin B is nephrotoxic and miltefosine may be nephrotoxic. Miltefosine may cause bleeding problems I have seen, and Amphotericin B may cause some sort of thrombocytopenia.”* (India, NP8)  *“Second thing, it is for the patient follow up, pharmacovigilance must be strengthened with these patients.”* (India, NP9) |
| Need for more frequent monitoring (NP) | *“There should be a difference in the follow-up between VL patients and co-infected patients. Frequency of follow-up should be more”* (India, NP9) |
| Accessibility barrier for monitoring (NP) | *“Definitely, coming back, I told you that accessibility is a very big issue in India, that they even have to travel 200 kilometers, something like that. […] Basic thing is that there should be more treatment centers for HIV/VL in tertiary care centers and those tertiary care centers should check that centers are integrated. If there is an integrated system, that will be the best.”* (India, NP9) |
| Need for monitoring of relapse (NP) | *“After completion of therapy we have to closely monitor, we have to closely follow up the patient for any sign or symptom of relapse.”* (India, NP8) |
| ***Research priorities*** | |
| Drug interactions (NP) | *“What is not known to us is that whether miltefosine can have some drug-drug interactions with some antiretroviral that are used, because we don’t know, we are not aware of it […] I think there is no information regarding this.” (India, NP1)* |
| Timing of administration of antiretroviral therapy (NP) | *“Should we start antiretroviral simultaneously with the antileishmanial treatment or one should wait for the treatment to be completed? 2 weeks-treatment with combination therapy then start of antiretroviral therapy? Because there is no guidance on this.” (India, NP1)* |
