# Supplementary material for: Stakeholders’ views and perspectives on treatments of visceral leishmaniasis and their outcomes in HIV-coinfected patients in East Africa and South-East Asia: A mixed methods study
Source: PLoS Negl Trop Dis. 2022 Aug 15;16(8):e0010624. doi: 10.1371/journal.pntd.0010624 (PMC9410553; doi:10.1371/journal.pntd.0010624)
Supplement: S5 Appendix — (DOCX) [file pntd.0010624.s005.docx]

**S5 Appendix:** Results of survey questions about implementation considerations, monitoring and evaluation, and research priorities

**1. Implementation considerations mentioned by survey participants**

- Staff
  - Training on diagnosis, treatment, adverse drug events, consideration of comorbid conditions, counselling
  - Sensitization for VL diagnosis in HIV patients
- Access to care
  - Availability of diagnostics, early identification of cases, availability of X-ray and sputum test to rule out tuberculosis
  - Availability of drug supply
  - Considerations for geographical location and cost concerns (e.g. need for travel compensation, wage loss compensation for the patient/family)
  - Availability of treatment at specialized centers who also have expertise in managing other complications of HIV infection (e.g. TB coinfection)
- Quality assurance
  - Cold chain maintenance
  - Storage conditions of the medications
- Guidance or implementation aids
  - Treatment protocol at facility, flow charts, treatment algorithms, SOP
  - Guidelines for HIV-VL relapse
- Coordination
  - Coordination between VL and HIV programs at national, state, district and block level
  - Formation of network between Bangladesh, India, Nepal and other South-Asian countries
- Increasing awareness among risk population (e.g. about co-infection, importance of treatment protocols, likely side effects); education to patient, family and community
- Setting up mechanisms to ensure that no discrimination or social stigma is generated during the treatments
- Individualized treatment approach
- Counselling
- Holistic care; taking into consideration other opportunistic infections, malnutrition status
- Psycho-social support
- Start of ART early in the illness; choice of ART (to avoid additive toxicities)
- Concern for immune reconstitution inflammatory syndrome (IRIS)
- Screening for pregnancy and contraceptive considerations (e.g. availability, considering injectable contraception)
- Need for testing of all VL patients for HIV; testing for testing of all HIV patients for VL in Kala-azar endemic areas
- Communication strategies to reach out to people with HIV

**2. Monitoring and evaluation suggestions mentioned by survey participants**

- What to monitor
  - Adverse events
  - Complications
  - Relapse
  - Adherence
  - Drug resistance
  - Other nosocomial infections
  - Nutritional status
  - Quality of life
  - CD4 count and viral load
  - Use of effective contraception (relevant to patients receiving miltefosine)
  - Adherence to guidelines from both HIV and VL program
  - Standardized monitoring and evaluation tools, case definitions, outcome indicators
  - Stock status of drugs
  - Zero-transmission Kala-azar Strategy
  - Recognition of PKDL (concern that all skin lesions are being labelled PKDL when in fact they may have been kala-azar with skin lesions)
- Monitoring logistics
  - Availability of laboratory for testing and monitoring
  - Non-invasive test of cure tests
  - Parasitological follow up (bone marrow examination, splenic aspiration)
  - Pharmacovigilance system,
  - Avoiding stigma
  - Patient confidentiality and privacy
  - Monitoring facilities at district hospital
  - Monitoring schedule, monitoring checklist
  - Proper record maintenance, patient file with daily clinical notes
  - Identified local institutions to play an active role in monitoring and evaluation (e.g. local medical colleges, institutes with a public health mandate), field level staff that are Kala-azar specific may be recruited like Kala-azar Technical Supervisors
  - Frequency of follow up may be advised
  - Timely and complete case reporting, number of cases
  - GIS mapping
  - Financial resources

**3. Research priority areas mentioned by survey participants**

- Disease epidemiology
  - Impact of VL on HIV progression to AIDS
  - Long term follow-up for the development of post-kala-azar dermal leishmaniasis
  - Social determinants of VL in HIV patients
- Diagnostic aspects
  - Need for better case definition
  - Diversities in clinical presentation
  - Diagnostic tests/tools
  - Screening HIV patients living in endemic areas for asymptomatic VL
- Prevention
  - Vaccine for VL
  - Prophylactic therapy (e.g. with liposomal amphotericin B or pentamidine)
  - Asymptomatic individuals and role of primary prophylaxis for HIV-VL (mentioned by one participant)
- Disease progression and prognosis
  - Causes of relapse
  - Biomarkers of relapse
  - Comparison of the two treatment alternatives in terms of long term prognosis
  - Predictors of good treatment outcome (e.g. level of viral load/HIV status, nutritional status, diet modification including protein restriction and diet rich in fatty acids, gender differences if any)
  - Time after co-infection that needs to be taken into account for declaring patients ‘cured’ of VL
  - Ways to prolong disease free period after treatment
  - Long term follow-up for development of PKDL, treatment of PKDL
  - Impact of VL on HIV progression/progression to AIDS
- Need for generation of additional data
  - Profiling of cases with other comorbidity and opportunistic infections
  - Survival index among treated cases
  - Comparison study between Asia and Africa for transmission dynamics
  - Data generation in newer foci areas, in countries where HIV/VL is not highlighted (e.g. Sudan): need to assess magnitude of the problem, need to assess effectiveness of treatments (i.e. comparative study between monotherapy and combination)
  - Prevalence of HIV among VL cases and vice versa
- Treatment-related issues
  - Alternative treatments (e.g. new VL treatment options)
  - Drug resistance
  - Drug interactions and anti-TB drugs
  - Methods to enhance compliance to treatment, knowledge, attitude and practices of patients regarding the course of treatment (including compliance)
  - Tolerance to the treatment according to age
- Immunology
  - Immunomodulators
  - Immune activation
  - Effect of combination therapy on immunity
- Relationship with HIV
  - Increase in CD4+ count and decrease in viral load after treatment
  - Use of antiretroviral therapy (e.g. which regimen of antiretrovirals is best to combine with anti-leishmanial drugs, time of ART start, role of ART in delaying relapse of VL, potential role of HIV medicines such as protease inhibitors)
- Health systems
  - Operational research aiming at explaining reasons for delays in providing care
  - Treatment services to migratory patients and supply chain management
- Social determinants of VL in HIV patients
- Co-infected individuals as reservoirs of Leishmania
- Treatment of pregnant women and effect on the unborn child
- Vector control and sand-fly behavior, use of long-lasting insecticidal nets (LLINs)
- Infectivity level of patients with co-infection compared to mono-infected patients
