# Supplementary material for: Stakeholders’ views and perspectives on treatments of visceral leishmaniasis and their outcomes in HIV-coinfected patients in East Africa and South-East Asia: A mixed methods study
Source: PLoS Negl Trop Dis. 2022 Aug 15;16(8):e0010624. doi: 10.1371/journal.pntd.0010624 (PMC9410553; doi:10.1371/journal.pntd.0010624)
Supplement: Attachment — Submitted filename: Response letter_20220107.docx [file pntd.0010624.s006.docx]

January 11, 2022

Prof. Mitali Chatterjee, Associate Editor, PLOS Neglected Tropical Diseases

Dr. Helen Price, Deputy Editor, PLOS Neglected Tropical Diseases

Re: “Stakeholders’ views and perspectives on treatments of visceral leishmaniasis and their outcomes in HIV-coinfected patients in East Africa and South-East Asia: a mixed methods study”

Dear Prof. Chatterjee and Dr. Price,

Thank you for the opportunity to revise and resubmit our above titled manuscript to PLOS Neglected Tropical Diseases. We thank you and the reviewers for taking the time to review the manuscript. We found the comments and suggestions very constructive and used them to improve our manuscript.

Kindly find below a point-by-point response to the reviewers’ comments and a description of the changes made to the manuscript.

We look forward to the outcome of the peer review of our revised manuscript.

Sincerely,

Elie A. Akl, MD, MPH, PhD

AUBMC, Department of Internal Medicine

P.O. Box: 11-0236

Riad-El-Solh Beirut 1107 2020

Beirut – Lebanon

Phone: 00961 1 350 000

[ea32@aub.edu.lb](mailto:ea32@aub.edu.lb)

**The reviewers’ comments are listed below in in regular font. Our responses to these comments are in bold. Extracts from the text are in italic font. We have indicated the sections where revisions have been made in our manuscript.**

**Reviewer 1**

The aim of the study is unclear. The design looks ok. A mixed study design and a variety of study participants were included. The conclusions could be expected to vary depending on the category of study participant. The sample size for qualitative study is ok. There are no ethical concerns.

**Thank you for the positive feedback regarding our study design. We agree that the conclusions could be expected to vary depending on the category of study participant, and we further highlight this in our discussion section. Please see below our response regarding the aim of the study.**

The figures particularly figure 3 should be revised.

**The Reviewer provides below more detailed comments on the figures. Accordingly, please refer to our below detailed replies with regards to the figures.**

Limitations of the study are mentioned. The authors have not officially made specific conclusions and recommendations. However, there is a hidden conclusion that for me appears to be unsubstantiated.

**It was not clear to us what the Reviewer is referring to with ‘hidden conclusion’. It would be helpful if this could be clarified.**

General comments:

This is an interesting manuscript that analyses outcomes, impact on equity, feasibility and acceptability of the treatment options in HIV co-infected VL patients.

For this manuscript to have impact on policy, a few issues need to be transparently discussed and to revisit the aim of this manuscript. Given that no effective treatment exists for such patients, I wonder why the authors chose to carry out comparison of two treatment regimens: combo and monotherapy? The report will have more impact if it can highlight the critical gaps in therapeutic options for HIV co-infected VL patients rather than comparing two regimens that are both sub-optimal.

**Thank you for raising this issue. We do agree with the reviewer regarding the critical gaps in therapeutic options. While the Reviewer suggests revisting the aim, we would like to clarify that, and as mentioned in the introduction of the manuscript, this study was conducted to support the World Health Organization (WHO)’ process to develop evidence-based region-specific recommendations on treatment regimens for VL in HIV-coinfected patients. The WHO guideline process requires the consideration of, in addition to the evidence on health effects, evidence on valuation of outcomes, and on the impact on equity, acceptability, and feasibility of the interventions being considered. Hence, our findings were intended to help inform the panel judgements when developing these specific recommendations on combination therapy vs monotherapy.**

**To emphasize the point about the ‘critical gaps in therapeutic options’, we do highlight in the introduction that *“treatment of VL in HIV–coinfected patients is constrained by reduced numbers of therapeutic options due to few clinical trials, ineffectiveness, or drug toxicity.”* Furthermore, we believe that this study still highlights critical gaps of the treatments in terms of other aspects, including equity, acceptability, feasibility, implementation considerations, monitoring and evaluation, and research priorities related to the treatments.**

As the NP stakeholders have identified relapse as the main feature of HIV-VL and the biggest challenge in treatment; valuations of outcome, impact on equity, feasibility and acceptability will be meaningful if there was a focus on the biggest challenge and how patients and stakeholders recognize the challenges and perceive public health constraints in this domain.

**Since one of the objectives of our study was to assess the valuation of outcomes of treatment, we had to include all outcomes that were of interest for the WHO panel.**

In a recent published report of a northern Ethiopia study, efficacy of total dose of 40mg/kg of ambisome monotherapy at day 29 was 50%, and the combination of Ambisome (30mg/kg total dose) and 28-day regimen of miltefosine resulted in 67% initial cure. At day 56 cure rate was 55% in the monotherapy arm, but 88% in the combo when treatment was extended by additional 28 days. Data on extended treatment is not available for the monotherapy, as it was not planned in the mentioned trial of northern Ethiopia. These findings show that the treatment of HIV co-infected patients will remain to be a challenge. Given that the monotherapy was horribly ineffective at the tested regimen, the comparative approach taken in this study does seem to be biased and in favour of the combination treatment obviously due to the better (but sub-optimal) efficacy. In the follow-up report of the above-mentioned study involving 54 patients, it was shown that the probability of relapse-free survival at one year was 50% (53% in 22 patients with baseline CD4 count > 200/µl hence not having secondary prophylaxis; and 46% in 29 patients with CD4 cells < 200/µl who were on secondary prophylaxis). It is important to avail such information to study participants (P & NP) to enable them to make a fair judgement and valuation. The fact that the NP stakeholders emphasized on the importance of the 6-month cure is indicative of the recognition of the challenges in treatment., i.e., the high risk of recurrent relapses and death. The premise (and context) for the qualitative and quantitative studies of patient outcomes should be clearer and well informed.

**We thank the reviewer for bringing this information to our attention. As mentioned above, the study addressed the therapeutic regimens (including length of treatment) considered by the guideline panel, in accordance with the WHO guidelines development process. This study was not intended to address the health effects (benefits and harms) of these regimens, but was intended to address the contextual factors, such as impact on equity, acceptability, and feasibility.**

**We would like to clarify that although we ask questions using a comparative approach, the comparison is not made with regards to comparative efficacy of interventions, but with regards to the impact on equity, acceptability, and feasibility. For example, a treatment could have high efficacy but not be acceptable to key stakeholders. Furthermore, the valuation of outcomes does not take into consideration (i.e., it is independent from) the specific interventions.**

Specific comments

1.	This study reveals that combination therapy was more feasible and acceptable than monotherapy mainly due to a shorter duration of hospitalization. Because the trial results are yet to be translated to policy, and the policy/guideline recommendations are yet to come; iIt is not yet clear how patients would be treated in the routine health settings using the combo or monotherapy to reveal which regimen has a shorter duration of hospitalization. The combo treatment regimen for South-East Asia and Eastern Africa regions are varied and necessitate the disaggregation of the data by geographic regions. In the case of the Eastern Africa region, the 28-day miltefosine in the combo (which will be 56 days when extended) is no shorter than the monotherapy that lasts 24 days (days 1,2,3,4,5,10, 17,24). In assessing acceptability, equity and feasibility comparing the combo with monotherapy; the tools (questionnaire) were not reinforced with specific questions that could help to substantiate claims on better acceptability, feasibility, and equity of the combo treatment regimen.

**Based on the information WHO received from the trialists (trial from India) and on the information in the published trial from Ethiopia, combination therapy was associated with less duration of treatment. Indeed, the duration of monotherapy treatment in the Eastern Africa region and elsewhere as recommended by WHO is 38 days (3–5 mg/kg daily or intermittently for 10 doses (days 1–5, 10, 17, 24, 31 and 38) up to a total dose of 40 mg/kg), which is longer than the 28 days duration of combination therapy. Further details can be obtained from the reference WHO Technical Report Series 949 (**[**https://apps.who.int/iris/handle/10665/44412**](https://apps.who.int/iris/handle/10665/44412)**). The study was conducted based on this information. The duration of admission or further treatment depends upon a case-by-case basis and upon a variety of factors. However, if indeed, the duration will be different when the recommendations are implemented, our findings need to be interpreted accordingly. Accordingly, we have added the following text to the discussion section:**

***“Also, if the duration of the combination in practice will be longer compared to the trial protocols, our findings need to be interpreted accordingly.”***

A second layer of questions should have been included why P/NP study participants believe a treatment regimen was more acceptable, more feasible, and more equitable. It would have been preferred if context was projected, and explicit information why combo was found to be more equitable, more acceptable, and more feasible was interrogated.

**Thank you for raising this issue. While the quantitative aspect of the study does not help in answering the “why” question, we believe that the qualitative part of the study allowed us to further understand this aspect. In appendix 3, we provide detailed explicit information from participants supporting our findings.**

Furthermore, looking carefully at the narrations of the results, equity was not an issue either in the combo or monotherapy. Similarly, concerning feasibility, the only issue was the contraindication in pregnant women. Prolonged hospitalization could be an issue of feasibility and acceptability, but only in South-East India. As mentioned above, the crux of the investigations in this report would preferably focus on the management of recurrent relapses, i.e., the need for management of the chronic conditions and quality of life including the need for secondary prophylaxis. To my opinion, the authors did not demonstrate the existence of consensus to support the claims of feasibility, equity and acceptance of the combo more than the monotherapy. It is important to recognize that even with the best treatment options available today, the expectancy of survival in HIV co-infected patients is short (3 years at maximum). Given this, the emphasis on assessing quality of life of patients would have helped to advocate for further studies on new approaches and avenues of intervention to improve quality of life and to prolong years of survival - as in the case of ARTs in HIV/AIDS patients.

**We thank the Reviewer for the excellent questions raised about the clinical care of patients co-infected with VL and HIV (e.g., management of recurrent relapses, management of the chronic conditions, secondary prophylaxis). As clarified earlier, none of these was the focus of the WHO guideline question, and consequently of our study.**

**We absolutely agree with the need to advocate for further studies, and we have added the following to the ‘implications for future research’ section under discussion:**

***“More generally, there is a need for studies assessing the impact of new approaches and interventions on quality of life in the population of interest.”***

2.	Table 1 gives 26 females. Does it mean the number of males was 151? The table does not give the figure for males.

**Thank you for highlighting this. The number of males is 148. We have added this to the table, and added a footnote clarifying that:**

***“Numbers do not always add to 177 due to missing data. Percentages represent valid percentages”*.**

3.	Figure 3 is presented in a biased way favouring the combo or focussing on combo (in a non-comparative way). For instance, the figure on acceptability is exclusive to combo (not showing rates for monotherapy). Only the feasibility component is presented in a comparative way. It is difficult to see from the figure the valuations about the monotherapy concerning acceptability and equity.

**Thank you for raising this important point. In the questionnaire, we had clarified to participants that the question was comparative. For example, the question on equity was phrased in the following way: “What would be the impact of combination therapy (as compared to monotherapy) on health equity?”**

**For further clarification, in addition to the title of the figure referring to *“combination therapy as compared to monotherapy”*, we have added for each factor the following underlined text:**

- **Impact on equity of combination therapy as compared to monotherapy**
- **Acceptability** **of combination therapy as compared to monotherapy**
- **Feasibility** **of combination therapy as compared to monotherapy**

**We also added the proportion of participants answering “varies” and “don’t know” (previously not displayed).**

4.	Figures 1,2, 3 lack title and legend.

**Thank you for highlighting this. As per PLOS NTD requirements, the titles of the figures are required to be inserted in the manuscript: “insert figure captions in manuscript text, immediately following the paragraph where the figure is first cited.” Hence, the titles of the figures can be found in the manuscript file, and are pasted below for the reference:**

- **Fig 1: Mixed methods design**
- **Fig 2: Valuation of each of the outcomes of interest**
- **Fig 3: Impact on equity, acceptability, and feasibility of combination therapy as compared with monotherapy**

**We did not include a legend for the figures because they were not required by the journal, and we believe that they are self-explanatory. However, we would be happy to include them if the editors prefer so.**

**Reviewer 2**

The paper appears to be good. But I think a few lines about ART and drug interactions need to be mentioned. Also tuberculosis is the most important opportunistic infection in India so a few lines on this treatment modality needs to be mentioned.

**We thank the Reviewer for raising this. WHO guideline on the treatment of VL in HIV-coinfected patients (in press) has already addressed these issues in details along with appropriate cross-reference with the recent launch (July 2021) of WHO Consolidated Guidelines on HIV Prevention, Testing, Treatment, Service Delivery and Monitoring. However, we have added following statement in the text**

**“*While timely VL treatment is essential for reducing morbidity and mortality of these patients, adequate management of HIV is also important (e.g., HIV testing, selection of antiretroviral therapy (ART), timing of ART and careful consideration of drug-drug interactions while selecting appropriate regimen), contributing most importantly to reducing risk of VL relapse”.***

**Reviewer 3**

Although the approach is interesting, I have a number of thoughts on the study. My main concerns are on the lack of participation from the East African setting, where the case numbers, challenges and level of expertise in VL-HIV is far higher than in the Indian setting where it is a relatively new phenomenon that is being treated/managed by a very limited group of stakeholders. Secondly, one of the most important parts of this study is the patient involvement. The numbers of patients interviewed (n=19) are really impressive and should have been enough to generate a wealth of evidence in what is one of the most under-described perspectives (I am assuming here that these were VL-HIV patients). However, the description of the methodology and subsequently the content and richness of the feedback presented in the manuscript does not seem to be adequately covered – recognising that the interviews were done over the phone – but this needs better description and explanation of what transpired and why this was not enough to get some clear thematics.

**We agree that one of the limitations of the study is a lower participation from the East African setting, and now acknowledge this, and explain it in our discussion section:**

***“Another limitation is that this study had lower participation from East Africa as compared to South-East Asia. This was mainly due to unavailability of email addresses of health personnel in East Africa, and a lower response rate from East Africa as compared to South-East Asia.”***

**In fact, 63 stakeholders were contacted for participation in the study (which was voluntary) but only 32 responded in spite of two automated reminders at two weeks’ interval. We now clarify this in the methods and results section:**

***“Based on obtained email addresses, the majority of invited participants were from South-East Asia (76%).”***

*“****Out of 260 invitations, we received 177 complete responses (overall response rate = 68%, response rate in South-East Asia = 72%, response rate in East Africa = 51%).”***

**We confirm that the interviewed patients were VL-HIV patients. We hypothesize in our discussion about how telephone interviews may have affected the richness of data obtained from patients (i.e., the reason why some patients were not very open about their views, even after probing).**

Introduction:

**Thank you for the below comments regarding the introduction section.**

•	Note that atypical disseminated leish is defined as a stage 4 defining illness rather than VL.

**We have replaced the citation to cite the global reference of WHO Technical Report Series 949, page 65, last paragraph (**[**https://apps.who.int/iris/handle/10665/44412**](https://apps.who.int/iris/handle/10665/44412)**): “Visceral leishmaniasis is an AIDS-defining condition and a valid entry point for starting antiretroviral treatment, irrespective of CD4+ count”.**

•	For example, in India, the percentage of Kala-azar (KA) cases tested for HIV increased from 47% in 2015 to 98% in 2020, representing an increase in the proportion of KA–HIV co-infected cases from 0.5% in 2015 to 4.1% in 2020 (4).

- I’m unsure about this reference, but this number on proportions tested in India is very wrong. In 2015, barely any VL patients were being tested for HIV (certainly <10%). And 98% testing rates in 2020…its highly unlikely. The limitations of KAMIS need to be at least alluded to (eg there is no zero-reporting for HIV testing).

**Regarding inaccuracy of HIV testing numbers in India for the past years; this has been taken from annual VL surveillance report from National Vector Borne Disease Control Programme submitted to WHO for publication in *weekly epidemiological record.* This is an available reference and validating accuracy of this information was beyond the scope of this article. Also examining quality and limitations of a national VL Health Management Indormation System of Ministry of Health of India is beyond our scope of survey and this article.**

•	This is a major challenge considering that L. infantum has different drug susceptibilities and less virulence than L. donovani and occurs at a lower CD4+ count.

- Unclear what is meant by ‘occurs at a lower CD4 count’? Both infections occur at low CD4 counts.

**We agree with the Reviewer that both infections occur at low CD4 counts. The sentence meant to highlight that L. infantum occurs at a lower CD4+ count as compared to L. donovani (according to the following reference:** [**https://www.who.int/leishmaniasis/resources/Leishmaniasis_hiv_coinfection5.pdf?ua=1**](https://www.who.int/leishmaniasis/resources/Leishmaniasis_hiv_coinfection5.pdf?ua=1)**, “Relapses due to L. donovani occur at higher CD4+ counts than is the case with L. infantum in Europe.”)**

•	The regimens consist of combination of liposomal amphotericin B infusion (at a dose of 5 mg/kg on days 1,3,5,7,9 and 11, up to a total dose of 30 mg/kg body weight) and oral miltefosine (100 mg in divided doses for 14 days in South-East Asia and 28 days in East Africa respectively)

- Note that the East African regimen is required to be repeated in many cases, so treatment duration is doubled. This should be checked and worded accordingly.

**In this paragraph, we are describing the standard recommended regimens as described in the WHO Technical Report Series 949 (**[**https://apps.who.int/iris/handle/10665/44412**](https://apps.who.int/iris/handle/10665/44412)**). However, we agree with the Reviewer that the duration of admission or further treatment depends upon a case-by-case basis and upon a variety of factors, and have added this information at the end of this paragraph:**

***“Generally, in this patient population, the duration of admission or further treatment depends upon a case-by-case basis and upon a variety of factors*** ***such as delay in knowing HIV status, delay in seeking VL treatment, other comorbidities and opportunistic infections”***

Methods:

•	It is worth explaining what a listserv is; most people will not know this

**Thank you for raising this issue. We have added an explanation of listserv: *“computerized list of names and e-mail addresses”***

•	Were all interviews conducted in English/in person? Who coded the transcripts and were these double coded/QA’d by another researcher? There needs to be more robust description of the qualitative methodology in general. I see this is in appendix 2 = the authors should move this into the main manuscript or dedicate more space to describe this in the text.

**Thank you for mentioning these important points.**

- **We had already included a sentence mentioning that interviews with patients were conducted in local language, and we have now added that interviews with non-patient stakeholders were conducted in English.**
- **Due to the COVID-19 pandemic and logistical issues, the interviews were not conducted in person, which is clarified into the manuscript: *“We interviewed participants over the telephone, or using an online meeting platform.”***
- **We have moved details from appendix 2 to the main text in order to provide a more robust description of the qualitative methodology (see data analysis section of semi-structured interviews section).**

•	More details on the interview method/framework with the patients is needed

**The Reviewer provides below more specific comments regarding missing details. Accordingly, please refer to our below detailed replies with regards to details related to patient interviews.**

Results:

•	Did the response split between Asia/Africa reflect the numbers of invitations sent? I.e what proportion were sent to participants from both regions? It’s a very Asia heavy participation, which is odd since the bulk of VL-HIV patients come from the African setting, as does the majority of evidence. Would be important to explain this.

**The response split does reflect the numbers of invitations sent, but also reflects the response rate (see above explanation). This also reflects on the distribution of interviews per region since we used the survey as a gateway to recruiting individuals for the interviews. As mentioned above, we now reflect this in our methods, results and discussion sections (where we provide two main reasons for the lower proportion of participants from East Africa). In addition, we would like to clarify that we are aware that in South Asia, e.g., India, the number of stakeholders is high due to the presence an elimination programme. This could explain why we received most email addresses and a massive response for survey participation from this region. We are also aware that HIV-VL is mainly a problem in Ethiopia and information from other East African countries is rudimentary and incomplete. This could be another reason for limited participation.**

•	I think it could be important to split the table so that we can see the characteristics of the participants from Asia and Africa separately. Particularly for Africa, this will allow the reader to contextualise the feedback.

**Thank you for this suggestion. We have split table 1 according to participants’ region and included it in the appendix.**

•	Table 1: Region: 175/177 are reported – where are the other 2 from? Some of the strata do not add to 177, please check and annotate for missing data (either in the table or in the legend)

**Thank you for raising this issue. We have added a footnote clarifying that *“Numbers do not always add to 177 due to missing data. Percentages represent valid percentages”.***

•	Semi structured interviews with patients – specify if these were VL-HIV patients, and which treatments they had been treated with previously

**Thank you for the suggestion. We specify under the section “study population and sampling” that *“We included the same stakeholder groups as in the survey study (referred to as “non-patient stakeholders”), as well as VL patients with HIV-coinfection.”* The treatments received were not of interest to this study. We have added a sentence clarifying that all patients under trial were offered for interview:**

***“Wherever possible, attempts were made to contact all VL patients under trial by the treating physicians. Patients were given information about this study, and were invited for the interview. Local principal investigators (DP and NM) then contacted patients who volunteered and expressed their willingness, obtained their informed consent and conducted the interviews.”***

•	The one equity issue raised is related to the contraindication of combination therapy in pregnancy.

Worth re-checking this phraseology – combo treatment is contraindicated in pregnancy, lactation and in women of childbearing age unwilling or unable to take/use contraception

**Thank you for this suggestion, we have added *“women of childbearing age who may not prevent pregnancy”* (as reported by one interviewee). Since interviewees did not bring up the issue of lactation, we did not include it in the results section.**

•	The patient interviews over the phone are mentioned only at this point, this should be in the methodology section. How were patients selected and invited to participate (also worth mentioning how they were consented). Were their interviews recorded? More details are needed for this component as this is one of the most interesting parts of the study – beneficiary impact perception is critical, but we need to better understand how this was done.

**Thank you for highlighting these issues. Under “data collection” section, which is under the “semi-structured interviews” section of the methods section, we mention the following: *“We interviewed participants over the telephone, or using an online meeting platform, and audio recorded the interviews.”* To be more specific, we changed the text into: *“We interviewed patients over the telephone, and non-patient stakeholders using an online meeting platform, and audio recorded the interviews”***

**As mentioned above, we have added the following text in order to provide more details about recruitment of patients:**

***“Wherever possible, all VL patients under trial were contacted by the treating physicians, were given information about this study, and were invited for the interview. Local principal investigators (DP and NM) then contacted patients who volunteered and expressed their willingness, and obtained their informed consent and conducted the interviews.”***

**The terminology “beneficiary impact perception” is not clear to us. We appreciate clarification so we could address it appropriately.**

•	Where we had insufficient information from the patients’ perspective, we indicated this as part of the above findings.

Is unclear what the authors refer to here, there is no mention of this in the previous text.

**Thank you for raising this issue. We have removed this sentence as we agree that it is unclear to the reader.**

•	Limitations – it seems that the biggest limitation here is that the African region was massively under-represented; this needs much more discussion on the causes and reasons, as otherwise the study is really only valid for the Indian/South Asian setting

**Please see above regarding acknowledging this in our limitations section and providing further explanation about proportion contacted and response rate.**

•	Again the listserv – it is worth describing which listservs the authors refer to and from where they were acquired. I am surprised that snowball sampling was not conducted – is there a reason why? The VL-HIV world is quite small, and this could have been a very good way of identifying stakeholders...

**Thank you for the comment. We provide the following clarification for the listserv:**

***“We obtained email addresses of stakeholders from databases available within WHO headquarter office, and contacted WHO regional offices for the African Region, Eastern Mediterranean Region and the Region for South-East Asia. The Ministry of Health of India, and WHO partners like Médecins Sans Frontières (MSF), Drugs for Neglected Diseases Initiative (DNDi) and Leishmaniasis East Africa Platform (LEAP) were also contacted.”***

**We agree that snowball sampling would have been a great approach, especially for the qualitative part of our study (interviews). As we already had the survey as our gateway to recruitment for the qualitative study, and as it resulted in a good number of interviewees, we did not need to resort to snowball sampling. Had the survey not yielded a good number of interviewees, we might have resorted to this sampling strategy.**

•	India phone numbers not being accurate = this is not surprising as people change sim cards very frequently in India.

**Thank you for mentioning this common issue in India. We agree as the interviewer confirmed that this could be due to variety of reasons, one being frequent change of sim cards.**
