# Supplementary material for: Stakeholders’ views and perspectives on treatments of visceral leishmaniasis and their outcomes in HIV-coinfected patients in East Africa and South-East Asia: A mixed methods study
Source: PLoS Negl Trop Dis. 2022 Aug 15;16(8):e0010624. doi: 10.1371/journal.pntd.0010624 (PMC9410553; doi:10.1371/journal.pntd.0010624)
Supplement: Attachment — Submitted filename: Response letter_20220511.docx [file pntd.0010624.s007.docx]

**Department of Internal Medicine**

**American University of Beirut**

May 11, 2022

Dr. Helen Price

Deputy Editor

PLOS Neglected Tropical Diseases

Re: “Stakeholders’ views and perspectives on treatments of visceral leishmaniasis and their outcomes in HIV-coinfected patients in East Africa and South-East Asia: a mixed methods study”

Dear Dr. Price,

Thank you for the opportunity to revise and resubmit our above titled manuscript to PLOS Neglected Tropical Diseases. We thank you and the reviewers for taking the time to review the manuscript, and for considering accepting this manuscript for publication.

Kindly find below a point-by-point response to the reviewers’ comments and a description of the changes made to the manuscript. It appears to us that Reviewer #1’s major concerns relate to the choice of regimens, which in fact represent the regimens considered by WHO for its recommendations. We have done our best to address those concerns while having to stick to the recommendations in the WHO guidelines.

We look forward to the outcome of the peer review of our revised manuscript.

Sincerely,

Elie A. Akl, MD, MPH, PhD

AUBMC, Department of Internal Medicine

P.O. Box: 11-0236

Riad-El-Solh Beirut 1107 2020

Beirut – Lebanon

Phone: 00961 1 350 000

[ea32@aub.edu.lb](mailto:ea32@aub.edu.lb)

**The reviewers’ comments are listed below in regular font. Our responses to these comments are in bold. Extracts from the text are in italic font. We have indicated the sections where revisions have been made in our manuscript.**

**Reviewer #1:**

1. Methods

The aim of the study still is unclear

**We address this issue in the related comment below.**

2. Results

Yes, results are clear. But one aspect of the investigations, i.e., the comparison of treatment duration, was carried out with a wrong premise. See general comments section below.

**We address this issue in the related comment below.**

3. Conclusions

The conclusion is unclear. See general comments section below.

**We address this issue in the related comment below.**

4. Summary and General Comments

 The manuscript has been improved substantially and given us a placated understanding of the problems in the treatment of HIV co-infected VL patients. However, I would like to invite the authors to address following:

    Concerning the AmBisome monotherapy treatment regimen, the authors have referred us to the WHO Technical Report Series 949, which indeed indicates treatments on days 1-5, 10,17,24, 31 and 38. This regimen has not been used in Eastern Africa endemic region, and the recommendation itself was very general and makes no reference to the endemic regions. At the time of the publication of Serie 949, no RCTs were conducted for VL patients with HIV co-infection.

    In the recent RCTs conducted in Eastern Africa (mainly Ethiopia), the miltefosine and AmBisome combo was used for minimum 28 days, and up to 56 days while monotherapy at 40mg/kg maximum dose was given in a treatment regimen lasting 24 days (days 1-5, 10, 17, and 24). It looks like the authors were not aware of this information, which had led them believe Ambisome monotherapy required longer duration of treatment. One then wonders if study participants were given the right information about treatment duration concerning HIV co-infection of VL. In one of the annexes/excel file/, responses like “Two drug is more feasible as it will require short duration of hospital stay” has been quoted. It is implied that use of two drugs will have the advantage of shorter hospital stay/treatment duration, which in fact is true in many conditions. However, as mentioned above the monotherapy had shorter duration contrary to what the authors considered. Thus, the section of this manuscript that

    dealt with duration of treatment is not valid.

**We thank the Reviewer for raising this issue and acknowledge the validity of the concern.**

**This study was conducted to inform the World Health Organization guideline expert panel that developed the region-specific recommendations for the treatment of VL in HIV-coinfected patients. The panel considered the following two treatment options, which are referenced in the introduction of the manuscript:**

- **In Eastern Africa**
  - **Liposomal Amphotericin B + miltefosine regimen: liposomal Amphotericin B (up to 30 mg/kg, at 5 mg/kg on days 1, 3, 5, 7, 9, 11) + miltefosine (100 mg/day for 28 days)**
  - **Liposomal Amphotericin B regimen: liposomal Amphotericin B (up to 40 mg/kg, at 5 mg/kg on days 1-5, 10, 17, 24)**
- **In South-East Asia**
  - **Liposomal amphotericin B + miltefosine regimen: liposomal amphotericin B (up to 30 mg/kg, at 5 mg/kg on days 1, 3, 5, 7, 9, 11) + miltefosine (100 mg/day for 14 days)**
  - **Liposomal amphotericin B regimen: liposomal Amphotericin B (up to 40 mg/kg, at 5 mg/kg on days 1-4, 8, 10, 17, 24)**

**The reviewer is expressing concern in relation to Ambisome monotherapy requiring longer duration of treatment specifically in Eastern Africa. Accordingly, instead of referring to combination therapy having a shorter duration regardless of the setting, we have edited the text into the following:**

***“combination therapy was more feasible and acceptable than monotherapy when associated with a shorter duration of hospitalization.”***

**We believe that this change would allow the reader to better interpret our findings according to the context (i.e., this finding would be applicable in contexts where the duration of combination therapy is shorter, such as South-East Asia). Also, in the revised version of our manuscript, we acknowledge the following:**

***“if the duration of the combination in practice will be longer compared to the trial protocols, our findings need to be interpreted accordingly.”***

**We would like to clarify that one of the challenges related to the two regimens being compared is that the relative durations of monotherapy and combination therapy vary by context. Indeed, there is no harmony in national guidelines and each of the endemic countries follows its own national protocol for HIV-VL treatment. Current National Leishmaniasis Control guidelines of East African countries namely Ethiopia, Kenya, Sudan, South Sudan, Somalia and Uganda, mention HIV-VL treatment with AmBisome monotherapy. As the reviewer mentions, in Ethiopia, the treatment protocol is liposomal amphotericin B at maximal dose of 40mg/kg on days 1 to 5 and then on the 10th, 17th and 24th day. However, in Somalia’s national leishmaniasis control guideline, the protocol is Lipid formulations (e.g., Liposomal Amphotericin B, Ambisome) infused at a dose of 3–5 mg/kg daily or intermittently for 10 doses on days 1–5, 10, 17, 24, 31 and 38, up to a total dose of 40 mg/kg (**[**https://www.who.int/leishmaniasis/burden/Guidelines_for_diagnosis_treatment_and_prevention_of_VL_in_Somalia.pdf**](https://www.who.int/leishmaniasis/burden/Guidelines_for_diagnosis_treatment_and_prevention_of_VL_in_Somalia.pdf)**). Accordingly, the duration of monotherapy would be higher than that of combination therapy in that context. Although the majority of HIV-VL coinfection occurs in Ethiopia, the WHO will have to take into account a larger perspective in the region. Indeed, the** **guideline in question is meant for all endemic countries of East Africa where L. donovani is the causative agent.**

    Further, the RCTs in Ethiopia have demonstrated poor efficacy of AmBisome monotherapy. Again, one wonders why this article we are reviewing had to deal with comparison of the inefficacious treatment with the combo treatment. In my original review, I asked what the aim of the study was concerning treatment options of HIV co-infected VL patients. I also raised a query about the lack of conclusions. The whole issue concerning feasibility, acceptability, valuation of outcomes and impact on equity makes sense if the interventions in place fulfil a minimum set of criteria related to patient outcomes (cure, relapse free state, improved quality of life). If I may ask the same question again, why was this study conducted given the fact that none of currently available treatments are effective enough? Is it because both regimens (mono and combo) are inefficacious, and that one needs to choose the better of these badly performing treatments?

**We thank the reviewer for raising this issue again, and for clarifying their comment in relation to our study aim/conclusion being unclear. As mentioned in our previous reply, we do agree with the reviewer regarding the critical gaps in therapeutic options for** **VL in HIV-coinfected patients. In fact, we agree that none of the monotherapy regimens, including liposomal amphotericin B monotherapy, is efficacious in East Africa. Also, there is lack of data from South Asia. However, the preferred choice of treatment in absence of any better option is liposomal amphotericin B 40 mg/kg. We would like to re-emphasize that this study was conducted to support the World Health Organization in developing region-specific recommendations on treatment regimens for VL in HIV-coinfected patients. These recommendations considered combination therapy vs monotherapy. This determined the aim of our study.**

**Reviewer #3:**

1. Methods

The responses below cover the author responses to Reviewer 3:

- The difference of participation from the two regions still needs further description, as does the implication on the overall validity of the study. It seems from the author reply that there were 32 stakeholders responded; however they then go on to describe 177 completed responses being received – this is confusing. It would important to be clear on the number of participants from each region at each stage of the study – please add c.

**Thank you for raising this issue. We have further clarified the number of participants from each region at each stage of the study, as per below:**

**For the survey:**

***“Based on obtained email addresses (n=260), the majority of invited participants were from South-East Asia (n=197, 76%).”***

***“Most participants were males (n=148, 85%), aged 31–50 years (n=115, 65%), from South-East Asia (n=142, 81%)”***

**For the interviews:**

***“We interviewed 19 patients (nine from Ethiopia and ten from India), and 10 non-patient stakeholders (******two from Ethiopia and eight from India). Non-patient stakeholders included four members of nongovernmental organizations, three physicians, two programme managers and one representative of a national programme.”***

- The authors are correct regarding what is written in the WHO technical report series 949; however it is common knowledge that this is in conflict with WHO HIV guidelines. Considering the scope of 949 is VL, written by a panel of VL experts (not HIV) it is of minimal relevance with regards to classification of VL as an AIDS defining condition since national programme HIV guidelines (and decisions to initiate ART) are based on the WHO HIV guidelines alone, and not the 949 report. This is reflected in all national HIV guidelines. This is well discussed in the following paper, and I think it is important to state this as this is a WHO-authored manuscript:

    van Griensven J, Ritmeijer K, Lynen L, Diro E (2014) Visceral Leishmaniasis as an AIDS Defining Condition: Towards Consistency across WHO Guidelines. PLoS Negl Trop Dis 8(7): e2916. <https://eur03.safelinks.protection.outlook.com/?url=https%3A%2F%2Fdoi.org%2F10.1371%2Fjournal.pntd.0002916&amp;data=04%7C01%7Cea32%40aub.edu.lb%7Ca23c8ef8fecd4571904e08da04fd88b4%7Cc7ba5b1a41b643e9a1206ff654ada137%7C1%7C1%7C637827784217032572%7CUnknown%7CTWFpbGZsb3d8eyJWIjoiMC4wLjAwMDAiLCJQIjoiV2luMzIiLCJBTiI6Ik1haWwiLCJXVCI6Mn0%3D%7C3000&amp;sdata=AOPb3Zg0MHs6gWYULOtorsfy8Epm9%2B1nyyuK4yIXKR8%3D&amp;reserved=0>

**Thank you for the clarification. We have removed “and is identified as a stage 4 acquired immunodeficiency syndrome (AIDS) defining condition” given the controversy in practice. We would like to note that in the updated WHO guideline document (unpublished), VL will be identified as clinical stage 4.**

- ‘We agree with the Reviewer that both infections occur at low CD4 counts. The sentence meant to highlight that *L. infantum* occurs at a lower CD4+ count as compared to *L. donovani* (according to the following reference: <https://eur03.safelinks.protection.outlook.com/?url=https%3A%2F%2Fwww.who.int%2Fleishmaniasis%2Fresources%2FLeishmaniasis_hiv_coinfection5.pdf%3Fua%3D1&amp;data=04%7C01%7Cea32%40aub.edu.lb%7Ca23c8ef8fecd4571904e08da04fd88b4%7Cc7ba5b1a41b643e9a1206ff654ada137%7C1%7C1%7C637827784217032572%7CUnknown%7CTWFpbGZsb3d8eyJWIjoiMC4wLjAwMDAiLCJQIjoiV2luMzIiLCJBTiI6Ik1haWwiLCJXVCI6Mn0%3D%7C3000&amp;sdata=VvvJ4uP7wifdiaFqoQQ0ihKp3%2F5O1Ocf%2FqgBTMVUl5A%3D&amp;reserved=0>, “Relapses due to *L. donovani* occur at higher CD4+ counts than is the case with *L. infantum* in Europe.”)’

    The reference refers to relapses, not primary infections. This needs to be corrected or removed.

**Thank you for the clarification. We have modified the sentence as follows:**

**“This is a major challenge considering that *L. infantum* has different drug susceptibilities and less virulence than *L. donovani* and that relapses due to *L. donovani* occur at a lower CD4+ count.”**

- "In this paragraph, we are describing the standard recommended regimens as described in the WHO Technical Report Series 949 (<https://eur03.safelinks.protection.outlook.com/?url=https%3A%2F%2Fapps.who.int%2Firis%2Fhandle%2F10665%2F44412&amp;data=04%7C01%7Cea32%40aub.edu.lb%7Ca23c8ef8fecd4571904e08da04fd88b4%7Cc7ba5b1a41b643e9a1206ff654ada137%7C1%7C1%7C637827784217032572%7CUnknown%7CTWFpbGZsb3d8eyJWIjoiMC4wLjAwMDAiLCJQIjoiV2luMzIiLCJBTiI6Ik1haWwiLCJXVCI6Mn0%3D%7C3000&amp;sdata=5RPYCHhV30gwY2sHh1zqR7Ka8Vn9qr3d%2Fr%2BbOC6r%2BDw%3D&amp;reserved=0>). However, we agree with the Reviewer that the duration of admission or further treatment depends upon a case-by-case basis and upon a variety of factors, and have added this information at the end of this paragraph"

    The 949 report does not include the 14-day regimen; this was first described by Mahajan et al in 2015. The 949 report was written in 2010. Same applies for the E African regimen. The 949 report on page 66 describes only one regimen: Lipid formulations infused at a dose of 3–5 mg/kg daily or intermittently for 10 doses (days 1–5, 10, 17, 24, 31 and 38) up to a total dose of 40 mg/kg are recommended (A). My point is that the East African regimen is repeated as per their publication in the majority of cases; and their conclusion recommends this.

    As a side point, I believe the South Asia study has now been published so should be referenced <https://eur03.safelinks.protection.outlook.com/?url=https%3A%2F%2Fpubmed.ncbi.nlm.nih.gov%2F35147680%2F&amp;data=04%7C01%7Cea32%40aub.edu.lb%7Ca23c8ef8fecd4571904e08da04fd88b4%7Cc7ba5b1a41b643e9a1206ff654ada137%7C1%7C1%7C637827784217032572%7CUnknown%7CTWFpbGZsb3d8eyJWIjoiMC4wLjAwMDAiLCJQIjoiV2luMzIiLCJBTiI6Ik1haWwiLCJXVCI6Mn0%3D%7C3000&amp;sdata=%2BF5ghx8%2F%2FHUfwLw9kA%2BjHTzDtW9o10i9iLlE6pse8BY%3D&amp;reserved=0>

**Thank you for the clarification. We completely agree with the reviewer and wish to clarify that by “standard recommended regimens”, we were referring more generally to the monotherapy regimen described in the paragraph. We have now added the reference to the 949 report when mentioning the monotherapy regimen. For the combination therapy regimen, we reference the trials, and have also added the reference to the India trial as provided by the reviewer.**

**We also now reflect the** **point of repeated regimen in the text as follows:**

**“Finally, if the duration of the combination in practice will be longer compared to the trial protocols (e.g.,** **due to repeated regimens), our findings need to be interpreted accordingly.”**

- The authors state in the reply that interviews conducted with patients were performed by telephone or using an on-line platform. Specifically, were the telephone calls with patients recorded (the authors seem to confirm this was the case later on in the responses, but it is important to be explicit here) and how were thematics and answers coded? Normally interviews require quite a lot of transcribing, how was this done and were any interviews double-coded? I can imagine that conducting this sort of interview over the phone must have been exceptionally challenging, and this should be explored in the text.

**Thank you for raising these issues. We have made the following edit to be more explicit about recording patient interviews:**

***“We interviewed patients over the telephone, and non-patient stakeholders using an online meeting platform, and audio recorded all interviews.”***

**With regards to coding and thematics, we have modified the text to provide additional details:**

***“We used the*** ***framework thematic analytical approach guided by the*** ***EtD framework, which consisted of the following seven stages (17):*** ***(1) transcription of the recordings verbatim (AEH and JK). Since interviews with patients were conducted in local language, the local investigators (DP and NM) translated interviews to English and transcribed them at the same time; (2) familiarization with the transcripts (AEH, CR and JK);*** ***(3) data indexing, which consisted of labeling each meaningful datum, initially applied to a few transcripts using the predetermined constructs of EtD framework (AEH, CR and JK). Texts that did not fit the predetermined constructs were also labeled; (4) discussion of labels leading to the creation of categories, forming the basis for the analytical framework (AEH, CR, JK and GHA); (5) application of the analytical framework to all transcripts, adding new labels that did not fit into the existing analytical framework when encountered (AEH, CR and JK); (6) charting and examining allocation of subcategories for each category, and reexamination of the labels that did not fit the existing analytical framework (AEH, CR and JK); (7) comparison and contrasting of categories and subcategories to finalize the results (AEH, CR, JK and GHA).”***

**Since we had the EtD framework as our starting point, we used a framework thematic analytical approach. In this qualitative approach, labels (codes) are grouped into categories, which are fitted under the framework.**

**As the reviewer mentioned, the interviews required extensive transcribing, which was conducted verbatim. We mention the transcription step in the text as follows:**

***“(1) transcription of the recordings verbatim (AEH and JK). Since interviews with patients were conducted in local language, the local investigators (DP and NM) translated interviews to English and transcribed them at the same time”***

**With regards to double-coding, all coders (AEH, CR and JK) first coded the same interview as part of a calibration exercise. The rest of the interviews were coded by one person. We have added these details to the text as follows:**

***“All coders (AEH, CR and JK) first coded the same interview as part of a calibration exercise.*** ***The rest of the interviews were coded by one person. Coders met to determine the analytical framework, and compare and contrast findings.”***

- "In addition, we would like to clarify that we are aware that in South Asia, e.g., India, the number of stakeholders is high due to the presence an elimination programme. This could explain why we received most email addresses and a massive response for survey participation from this region."

    I am not sure I agree with this argument. Regardless of the elimination programme,  the numbers of individuals involved with management and treatment of VL-HIV patients in India is extremely small – there are basically two centres that treat these patients (Kolkata and RMRI, Patna), who have been treating patients for the last 5 years or so. On the contrary, there is a vast range of decades worth of experience of VL/HIV in Ethiopia and Sudan, and as you can see from the attendance of meetings and consortiums (take ASCEND recently, for example) there are a wide range of stakeholders in the East African setting ranging from NGO, governmental, academic and beyond. Information and sources are not rudimentary; there are clear individuals and organisations that could have been approached to get a wider buy in when it became obvious that the initial sample framework for East Africa was inadequate.  I think it is incorrect for the authors to explain this in this way; it seems rather

    that there was a greater effort made (unintentionally perhaps) for South Asia than for East Africa. This is important to self-reflect on, as it cuts to the core and validity of the messages and outcomes.

**The reviewer makes an important point in relation to underrepresentation of the East African setting. This is despite our efforts to obtain details of stakeholders from all possible sources in order to ensure representation from different geographical areas. As we have mentioned and added to the manuscript in the previous revision, we obtained email addresses of stakeholders from databases available within WHO headquarter office, and contacted WHO regional offices for the African Region, Eastern Mediterranean Region and the Region for South-East Asia. The Ministry of Health of India, and WHO partners like Médecins Sans Frontières (MSF), Drugs for Neglected Diseases Initiative (DNDi) and Leishmaniasis East Africa Platform (LEAP) were also contacted. We would also like to mention that ASCEND programme is a donor and does not implement VL activities and has single representatives in these countries. Nonetheless, ASCEND representatives and their implementing partners also participated in the study. As per the protocol of the study, participation was voluntary and anonymous. To increase the response rate, we sent two automated reminders for the survey after 7 and 13 days.**

**Following our last revision, we now clearly acknowledge the reviewer’s concern in the text, as we state the following under our limitations:**

***“Another limitation is that this study had lower participation from East Africa as compared to South-East Asia.”***

**Also, following the reviewer’s above comment that regardless of the elimination programme, the numbers of individuals involved with management and treatment of VL-HIV patients in India is extremely small, we have removed the sentence: *“Also, there are multiple stakeholders in South Asia due to the VL elimination initiative.”***

- “Wherever possible, attempts were made to contact all VL patients under trial by the treating physicians. Patients were given information about this study, and were invited for the interview. Local principal investigators (DP and NM) then contacted patients who volunteered and expressed their willingness, obtained their informed consent and conducted the interviews.”

    So if I understand correctly, all the VL-HIV patients were those that had previously participated in the phase 3 clinical trial on which the guideline change is based? This needs to be made explicit in the manuscript, and median time from treatment reported as the potential impact of recall bias should be clear, since this study was conducted long after the conclusion of the clinical trials. If correct, this is both a strength and a weakness for the study. A strength as this reflects directly patients who had either one of the treatments – and it will be critical to describe the proportions of those interviewed who received which treatment (eg. If all the patients received one of the treatment arms, they would not be a meaningful qualitative comparison). On the other side, all of these patients received treatment under clinical trial conditions, as such were admitted for the duration of treatment in hospital and receiving a level of care that would not be seen under routine conditions. These should be discussed in the limitations.

**We confirm that all the VL-HIV patients were those that had previously participated in the phase 3 clinical trials. We have further made this explicit in the manuscript:**

***“Physicians at the two treating centres in Ethiopia and India where the clinical trials were conducted (7, 8) helped with patient recruitment, while ensuring voluntary participation, confidentiality and anonymity. Wherever possible, attempts were made to contact all VL patients under trial by the treating physicians.”***

**We also provided the number of patients from each arm:**

***“Eight patients were from the combination therapy arm, and 11 were from the monotherapy arm.”***

**We understand the reviewer’s point with regards to recall bias. However, we believe that it is not very relevant to this study and should have not affected our results for the following reasons: 1- the part of the study that addresses valuation of treatment outcomes of HIV-VL relates to the disease in itself and is not dependent on the choice or receipt of treatment; 2- for the part of the study that addresses the interventions being compared, we made sure to explain what the two treatment regimens entailed in terms of drugs (e.g., 1 vs 2 drugs), durations, mode of administration (e.g., IV vs oral), and contraindications to the patients. This also applies to the fact that patients received treatment under clinical trial conditions, since we were not asking patients specifically about their experience with the type of care received. Nonetheless, we are happy to expand on these issues if the editor prefers so.**

- "The terminology “beneficiary impact perception” is not clear to us. We appreciate clarification so we could address it appropriately"

    Beneficiary impact perception is the perspective of the beneficiary (i.e. the patient with VL-HIV). I mean the purpose of interviewing this group was to determine their perspectives on the treatments they received – and the impact of the treatment on them. This is a much under-explored area in clinical trial research, and I commend the authors for exploring this – but they should try and go deeper into this as it is really important, especially as the number of patients (19, I believe) is really a huge sample size for such work. The authors state that there was insufficient information from the patient’s perspective – this simply cannot be the case with such a sample size unless there was something fundamentally wrong or lacking with the interview methodology used. If this is the case, that is fine, but it needs to be explained in the text.

**Thank you for clarifying the terminology “beneficiary impact perception.”**

**We have reflected further amongst the author group about our interview technique. We believe that, especially in this patient population, openness should be more during telephone interviews as there is no direct contact with the patient. Indeed, telephone interviews further conserved confidentiality and anonymity of patients.** **We believe that interviews through home visits would have been limited by hesitancy in expression of history and illness by the patient because of their family and the socially controlled environment in villages. We hypothesize that patients were not open because of the** **stigma and discrimination associated with their condition, and have added this in our discussion section. This is despite our efforts to probe participants.**

***“One limitation of the study is that some patients were not very open about their views, even after probing. This could be due to patients being uncomfortable with discussion due to stigma and discrimination associated with their condition (6). On that note, the use of telephone interviews further conserved confidentiality and anonymity of patients.”***

- "We agree that snowball sampling would have been a great approach, especially for the qualitative part of our study (interviews). As we already had the survey as our gateway to recruitment for the qualitative study, and as it resulted in a good number of interviewees, we did not need to resort to snowball sampling. Had the survey not yielded a good number of interviewees, we might have resorted to this sampling strategy."

    I find this response confusing still. The authors accept that there were insufficient responses from the East African region, but then state here that a ‘good number of interviewees’ were identified… Clearly, the method of participant sampling from East Africa was inadequate as it failed, so this should be expressed/explained.

**Thank you for the clarification. We now clearly acknowledge in the limitations of our study that the East African setting was underrepresented, as follows:**

***“Another limitation is that this study had lower participation from East Africa as compared to South-East Asia”***

**Also, we now further reflect on our sampling method and acknowledge its limitations in the discussion section:**

***“As we used the survey as our gateway to recruitment for the qualitative study, lower participation from the East African region in the survey study also reflected on the interviews.”***

2. Summary and General Comments

The answers are generally ok, but honestly it feels like the referencing is quite sloppy, and the underlying knowledge of the subject material limited, which is surprising since the author list includes some of the major players in VL. This sort of study -and I really do refer to the patient perspectives rather than the non-patient perspectives (which one could probably guess the answers and in my opinion reveals nothing novel), is critically important and I feel that the authors have really under-sold the study by not focusing much more on the outcomes of the patient interviews, or at least explaining why they were not able to generate decent evidence.

    More generally, I think the authors need to be clear and transparent on the failure to include adequate sampling from East Africa, and how that impacts the validity of the results.

**We thank the reviewer for raising important issues. We have clarified the use of certain references and addressed the reviewers’ concerns about them in the above sections. We agree with the reviewer about the importance of bringing to light the patient perspective. We now reflect further on why patients were not open about their views, despite our efforts to probe. We clearly state this as a limitation. Also, we now further reflect on underrepresentation from the East African setting.**
