# Supplementary material for: Stakeholders’ views and perspectives on treatments of visceral leishmaniasis and their outcomes in HIV-coinfected patients in East Africa and South-East Asia: A mixed methods study
Source: PLoS Negl Trop Dis. 2022 Aug 15;16(8):e0010624. doi: 10.1371/journal.pntd.0010624 (PMC9410553; doi:10.1371/journal.pntd.0010624)
Supplement: Attachment — Submitted filename: Response letter_20220615.docx [file pntd.0010624.s008.docx]

**Department of Internal Medicine**

**American University of Beirut**

June 14, 2022

Dr. Helen Price

Deputy Editor

PLOS Neglected Tropical Diseases

Re: “Stakeholders’ views and perspectives on treatments of visceral leishmaniasis and their outcomes in HIV-coinfected patients in East Africa and South-East Asia: a mixed methods study”

Dear Dr. Price,

Thank you for the opportunity to revise and resubmit our above titled manuscript to PLOS Neglected Tropical Diseases. We thank you and the reviewers for taking the time to review the manuscript, and for considering accepting this manuscript for publication.

Kindly find below a point-by-point response to the reviewers’ comments and a description of the changes made to the manuscript. We would like to note that some of the reviewers’ comments relate to the patient’s past experiences (e.g., what drug they received under trial, the fact that they were treated under clinical trial conditions). Since the objective of the study is to explore the views of patients (and of other stakeholders) in relation to their preferences in terms of two treatment modalities and not to their past experiences, we believe that past experiences should not have biased the patient’s responses. As we clarify below, trained interviewers made sure to thoroughly explain the two treatment modalities to patients, while highlighting distinguishing characteristics (e.g., the fact that one is a combination of two drugs, route of administration, possibility of additional side effects, duration, and contraindications). It was based on this explanation that patients were asked about their preferences.

We look forward to the outcome of the peer review of our revised manuscript.

Sincerely,

Elie A. Akl, MD, MPH, PhD

AUBMC, Department of Internal Medicine

P.O. Box: 11-0236

Riad-El-Solh Beirut 1107 2020

Beirut – Lebanon

Phone: 00961 1 350 000

[ea32@aub.edu.lb](mailto:ea32@aub.edu.lb)

**The reviewers’ comments are listed below in regular font. Our responses to these comments are in bold. Extracts from the text are in italic font. We have indicated the sections where revisions have been made in our manuscript.**

Reviewer #3: Thank you to the authors, the manuscript is much improved and acceptable for publication with the following slight adjustments:

**We thank the reviewer for their time to review our manuscript and for the insightful feedback.**

1) Participation from East Africa:

The authors still insist that the issue was of uptake; this is simply not true. The issue was that only 24% of invitations to participate were to East African stakeholders. In its worst interpretation, the current wording could be interpreted as a lack of interest from East African stakeholders, which is untrue (and honestly abit offensive) when this was rather a failure of methodology, and should be recognised as such. I suggest changing the limitation sentences to:

‘’Another limitation is that this study invited lower participation from East Africa as compared to South-East Asia.”

AND

“As we used the survey as our gateway to recruitment for the qualitative study, the substantially fewer invitations sent to East African participants in the survey study also reflected on the interviews

As a side note - ASCEND is a much bigger and successful programme in East Africa, where there are far more than one representative per country. Again, this response suggests an overly South Asian focus from the researchers..

**We thank the reviewer for the suggested edits. It is clear that we have a different interpretation than the reviewer regarding the reason for the low number of responses in East Africa. To avoid further back and forth, we opted not to comment on the reason for the low number of participants from East Africa, and restrict ourselves to objectively stating that the number of participants was low, as follows:**

***‘’Another limitation is that this study had*** ***a smaller number of participants from*** ***East Africa as compared to South-East Asia.”***

**Accordingly, we have deleted the sentence about how the recruitment for the survey reflected on the participation in the interviews.**

2) Patients responses:

Suggest making this more explicit and better worded:

Change:

Physicians at the two treating centres in Ethiopia and India where the clinical trials were conducted (7, 8) helped with patient recruitment, while ensuring voluntary participation, confidentiality and anonymity. Wherever possible, attempts were made to contact all VL patients under trial by the treating physicians.”

To:

“At the two treatment centres where the clinical trials were conducted, physicians attempted to contact all the patients who had participated in the trials, while ensuring voluntary participation, confidentiality and anonymity,”

**We thank the reviewer for the suggested edits. We have clarified the information as below:**

***“******At the treatment centre where the clinical trial in Ethiopia was conducted,*** ***physicians attempted to contact all the patients who had participated in the trial, while ensuring voluntary participation, confidentiality, and anonymity.*** ***A similar process was followed in India, except that a random sample of patients was contacted.”***

3) Recall Bias:

I disagree with the authors on this point. Even the patients recollection of their experiences with the disease will be impacted by recall bias, and its important from a comparison perspective also; eg. If the median duration was 5 years in Ethiopia vs 6 months in India, it is important to present this so appropriate interpretation on the integrity of the responses can be made. To this effect, please include the median and IQRs of the time-from-treatment to interview for each region.

**Thank you for raising this issue. We agree with the reviewer that had the objective of the study been to assess patients’ past experiences, the recollection of these would be impacted by recall bias. However, we would like to clarify that the objective of the study was to explore the views of patients in relation to their preferences and not to their past experiences of which type of treatment (monotherapy or combination therapy) improved their condition. Hence, no recollection of experiences was required from patients. Trained interviewers made sure to thoroughly explain the two treatment alternatives to patients, while highlighting distinguishing characteristics (e.g., the fact that one is a combination of two drugs, route of administration, possibility of additional side effects, duration, and contraindications). It was based on this explanation that patients were asked about their preferences. Hence, we believe that time-from-treatment to interview is not relevant.**

With regards to treatment, I find it somewhat unrealistic that the physicians were able to explain the ‘other’ treatment to the patients, and then elicit a meaningful opinion from them on which one they feel is better. Its also not possible to detach patient’s attitudes towards the treatments they received and the way they received it – so the fact that they were all treated as inpatients in both countries is important, and is a limitation on the validity of these perspectives in the real world setting where ambulatory treatment is likely to be encouraged, and where the standard of care/follow up/support is going to be far lower in the non-clinical trial context.

My suggestion is to briefly expand on this area; the methodology that the authors have used is extremely valuable and rare in NTD research, and all learning points and evidence should be presented accordingly – as I said in my previous review, the patient feedback is by far the most interesting and novel component of this research.

**We thank the reviewer for the positive feedback about the value of our** **methodology, and for the suggestion. We would like to clarify that, as stated in the manuscript, physicians helped with recruitment. However, it was trained interviewers from the author team who conducted the interview. The interviewers made sure to thoroughly explain both treatment alternatives to the patients, while highlighting distinguishing characteristics (e.g., the fact that one is a combination of two drugs, route of administration, possibility of additional side effects, duration, and contraindications). It was based on this explanation that the interviewers asked patients about their preferences.**

**Accordingly, we believe that the way patients were treated and whether they received monotherapy or combination therapy do not affect their views. Indeed, patients were not asked about their past experiences of which treatment was better (monotherapy or combination therapy). The objective was to explore patient’s preferences in relation to two treatment alternatives that were thoroughly explained to them (one option consisting of an injection vs one option consisting of injection plus oral medication for a certain number of days).**

4) ‘Openness more in telephone interviews’

This concept is surprising to me, and it would be good if the authors can provide some references to support this. In my understanding, for sensitive matters that are significant events, participants feel much more comfortable being interviewed in person, in private, where both a rapport can be built and privacy can be guaranteed. Typically, this done by inviting participants to attend a ‘safe’ place of interview separate to the residence (with travel and wage loss compensation paid). Over the phone, there is no guarantee of privacy, as the interviewer does not know the situation of the interviewee who may not be in a position to express that they are able to talk freely.

I would suggest that the statement “On that note, the use of telephone interviews further conserved confidentiality and anonymity of patients” is removed from the manuscript unless there is clear evidence to support this supposition. I suspect that the reason why patients were not open on the telephone was precisely because it is very difficult to build a rapport over the phone for sensitive subjects, and this should be recognized in the manuscript text as a potential limitation/cause of the poor result yield.

**We thank the reviewer for raising these points. While they seem reasonable, there is also literature supporting the value of telephone interviews to collect data with marginalized populations, including data related to sensitive subjects. We include references to this literature at the end of this letter. We would also like to clarify that the interviewers were trained on interviewing skills and did their best to establish rapport at the beginning of the interview by clearly describing the aims of the study, and issues related to voluntariness and confidentiality. All patients agreed to have the interviews recorded and no hesitation or concerns were sensed by the interviewers. This supports the fact that telephone interviews were positively received by the patients.**

**Since we have a different point of view than the reviewer with regards to the use of telephone interviews in this specific study, we have removed the controversial sentence:**

***“On that note, the use of telephone interviews further conserved confidentiality and anonymity of patients.”***

**We now replace it with the following statement:**

***“We are uncertain whether the use of face-to-face interviews would have yielded more in-depth information. However, they were not feasible given that this study was conducted during the COVID-19 pandemic.”***

**References related to the use of telephone interviews**

- **Drabble, L., Trocki, K. F., Salcedo, B., Walker, P. C., & Korcha, R. A. (2016). Conducting qualitative interviews by telephone: Lessons learned from a study of alcohol use among sexual minority and heterosexual women. *Qualitative Social Work*, *15*(1), 118-133.**
  - **Study design: qualitative interviews conducted over the telephone and exploring the lives and experiences of sexual minority and heterosexual women, including use of alcohol and drugs, social relationships, identity, and past traumatic experiences.**
  - **“Our experience affirms the both the viability and value of telephone interviews as a method for collecting rich narrative data with women from marginalized populations, including data related to sensitive subjects.”**
  - **“Interviewees provided rich descriptions of their life experiences, including experiences related to sensitive topics, such as sexual identity, alcohol or other drug use, and traumatic experiences.”**
- **Mealer, M., & Jones, J. (2014). Methodological and ethical issues related to qualitative telephone interviews on sensitive topics. *Nurse researcher*, *21*(4).**
  - **“Qualitative telephone interviews are a valuable method of collecting information on sensitive topics.”**
- **Ward, K., Gott, M., & Hoare, K. (2015). Participants’ views of telephone interviews within a grounded theory study. Journal of advanced nursing, 71(12), 2775-2785.**
  - **Overall, participants reported a positive experience of telephone interviewing. One identified theme was “not being judged or feeling inhibited”.**
- **Block, E. S., & Erskine, L. (2012). Interviewing by telephone: Specific considerations, opportunities, and challenges. *International journal of qualitative methods*, *11*(4), 428-445.**
  - **“May be more effective when the need for anonymity is high.”**
